# Age-induced nitrative stress decreases retrograde transport of proNGF via TrkA and increases proNGF retrograde transport and neurodegeneration via p75^NTR^

**DOI:** 10.3389/fnmol.2023.1241420

**Published:** 2023-11-13

**Authors:** Erika Kropf, Arman Shekari, Sama Jaberi, Anish Puri, Chengbiao Wu, Margaret Fahnestock

**Affiliations:** ^1^Neuroscience Program, McMaster University, Hamilton, ON, Canada; ^2^Department of Neurosciences, University of California San Diego, La Jolla, CA, United States; ^3^Department of Psychiatry and Behavioural Neurosciences, McMaster University, Hamilton, ON, Canada

**Keywords:** nitrative stress, pro nerve growth factor, basal forebrain cholinergic neurons, axonal transport, TrkA, p75^NTR^, aging, *in vitro*

## Abstract

**Introduction:**

Axonal transport of pro nerve growth factor (proNGF) is impaired in aged basal forebrain cholinergic neurons (BFCNs), which is associated with their degeneration. ProNGF is neurotrophic in the presence of its receptor tropomyosin-related kinase A (TrkA) but induces apoptosis via the pan-neurotrophin receptor (p75^NTR^) when TrkA is absent. It is well established that TrkA is lost while p75^NTR^ is maintained in aged BFCNs, but whether aging differentially affects transport of proNGF via each receptor is unknown. Nitrative stress increases during aging, but whether age-induced nitrative stress differentially affects proNGF transport via TrkA versus p75^NTR^ has not yet been studied. Answering these questions is essential for developing an accurate understanding of the mechanisms contributing to age-induced loss of proNGF transport and BFCN degeneration.

**Methods:**

In this study, fluorescence microscopy was used to analyze axonal transport of quantum dot labeled proNGF in rat BFCNs *in vitro*. Receptor specific effects were studied with proNGF mutants that selectively bind to either TrkA (proNGF-KKE) or p75^NTR^ (proNGF-Δ9-13). Signaling factor activity was quantified via immunostaining.

**Results:**

Young BFCNs transported proNGF-KKE but not proNGF-Δ9-13, and proNGF transport was not different in p75^NTR^ knockout BFCNs compared to wildtype BFCNs. These results indicate that young BFCNs transport proNGF via TrkA. *In vitro* aging increased transport of proNGF-Δ9-13 but decreased transport of proNGF-KKE. Treatment with the nitric oxide synthase inhibitor L-NAME reduced retrograde transport of proNGF-Δ9-13 in aged BFCNs while increasing retrograde transport of proNGF-KKE but did not affect TrkA or p75^NTR^ levels. ProNGF-Δ9-13 induced greater pro-apoptotic signaling and neurodegeneration and less pro-survival signaling relative to proNGF-KKE.

**Discussion:**

Together, these results indicate that age-induced nitrative stress decreases proNGF transport via TrkA while increasing proNGF transport via p75^NTR^. These transport deficits are associated with decreased survival signaling, increased apoptotic signaling, and neurodegeneration. Our findings elucidate the receptor specificity of age-and nitrative stress-induced proNGF transport deficits. These results may help to rescue the neurotrophic signaling of proNGF in aging to reduce age-induced loss of BFCN function and cognitive decline.

## 1. Introduction

Basal forebrain cholinergic neurons (BFCNs) are critical for learning, memory, and attention (Mesulam et al., 1983; Baxter and Chiba, 1999; Sarter and Parikh, 2005; Ballinger et al., 2016). Their highly branched axonal projections provide the primary source of cholinergic innervation throughout the cortex and hippocampus (Mesulam et al., 1983; Baxter and Chiba, 1999; Ballinger et al., 2016). BFCNs are extremely vulnerable during aging, as aged neurons exhibit loss of function, reduced axonal length, diminished hippocampal innervation, and loss of synapses (Colom, 2006; Ypsilanti et al., 2008). Such BFCN vulnerability is associated with age-related cognitive decline (Colom, 2006; Ypsilanti et al., 2008; Fahnestock and Shekari, 2019; Shekari and Fahnestock, 2021).

Nerve growth factor (NGF) is essential for the survival and function of BFCNs (Hefti, 1986; Williams et al., 1986; Hartikka and Hefti, 1988; Hatanaka et al., 1988; Lapchak and Hefti, 1991; Friedman et al., 1993; Koliatsos et al., 1994; Fahnestock and Shekari, 2019). NGF is not produced by BFCNs, so it must be retrogradely transported from BFCN target areas in the cortex and hippocampus to BFCN cell bodies in the basal forebrain (Korsching et al., 1985). NGF exerts neurotrophic function by binding to its high-affinity receptor, tropomyosin-related kinase A (TrkA) (Kaplan et al., 1991; Frade and Barde, 1998). NGF-induced activation of TrkA induces neurite outgrowth and pro-survival signaling by activation of the Ras-mitogen activated protein kinase-extracellular signal regulated kinase (Ras-MAPK–ERK), phosphatidylinositol-3-kinase (PI3K)-Akt, and phospholipase C-gamma (PLC-γ) signaling pathways (Kim et al., 1991; Vetter et al., 1991; Klesse et al., 1999; Kaplan and Miller, 2000). NGF also recognizes the pan neurotrophin receptor, p75^NTR^, with low affinity (Frade and Barde, 1998). In the presence of TrkA, p75^NTR^ increases the affinity of NGF for TrkA, enhancing its neurotrophic effects (Mahadeo et al., 1994). However, when activated in the absence of TrkA, p75^NTR^ inhibits neurite outgrowth and activates apoptotic signaling molecules such as cJun N-Terminal kinase (JNK) (Frade and Barde, 1998; Kaplan and Miller, 2000; Roux and Barker, 2002).

In the brain, NGF is detectable only as its precursor form, proNGF (Fahnestock et al., 2001), which is retrogradely transported similarly to mature NGF in peripheral and central nervous system neurons (De Nadai et al., 2016; Di Matteo et al., 2017; Shekari and Fahnestock, 2019). The biological activity of proNGF is dependent on the levels of its receptors, TrkA and p75^NTR^ (Lee, 2001; Masoudi et al., 2009). Although proNGF has a higher affinity for p75^NTR^ than for TrkA (Lee, 2001; Clewes et al., 2008), it retains neurotrophic activity when TrkA is present (Fahnestock et al., 2004a,b; Clewes et al., 2008; Masoudi et al., 2009; Ioannou and Fahnestock, 2017). Similar to mature NGF, proNGF binding to TrkA induces neurotrophic signaling, cell survival, and neurite outgrowth (Saboori and Young, 1986; Chen et al., 1997; Fahnestock et al., 2004b; Buttigieg et al., 2007; Clewes et al., 2008; Masoudi et al., 2009; Ioannou and Fahnestock, 2017). However, proNGF loses neurotrophic function and induces apoptotic signaling via p75^NTR^ when TrkA is reduced or absent (Lee, 2001; Beattie et al., 2002; Harrington et al., 2004; Nykjaer et al., 2004; Fahnestock et al., 2004a; Masoudi et al., 2009; Ioannou and Fahnestock, 2017).

Age-related loss of BFCN function and the associated cognitive decline may be caused by loss of proNGF transport to the basal forebrain (Cooper et al., 1994; Fahnestock and Shekari, 2019). NGF-immunoreactive material, later shown to be proNGF, accumulates in the aged cortex and hippocampus, while these levels are reduced in the aged basal forebrain (Scott et al., 1995; Peng et al., 2004; Al-Shawi et al., 2007; Terry et al., 2011). BFCNs aged *in vitro* also exhibit deficits in retrograde transport of proNGF (Shekari and Fahnestock, 2019). Furthermore, proNGF receptor levels are altered with aging. While TrkA mRNA and protein levels are reduced in BFCNs aged both *in vitro* and *in vivo*, levels of p75^NTR^ remain constant or increase (Koh et al., 1989; Cooper et al., 1994; Terry et al., 2011; Shekari and Fahnestock, 2019). Despite the well characterized receptor imbalance in proNGF receptor levels that occurs with age, it is unclear whether retrograde transport of proNGF via each of these receptors is selectively altered.

Nitrative stress, a subset of oxidative stress characterized by high levels of reactive nitrogen species, may contribute to age-induced proNGF transport deficits (Ozcan and Ogun, 2015; Kropf and Fahnestock, 2021). Reactive nitrogen species accumulate in the aged brain (Chakravarti and Chakravarti, 2017) and may contribute to proNGF transport deficits by inducing tau nitration and oligomerization, interfering with tau’s ability to interact with and stabilize microtubules (Zhang et al., 2005). Nitration of microtubules may also disrupt the association between retrograde axonal transport proteins and microtubules (Eiserich et al., 1999), which may contribute to the deficits observed in retrograde transport of proNGF in aged BFCNs. Reactive nitrogen species cause nitration of tubulin, preventing the interaction between tubulin and kinesin motors (Stykel et al., 2018). Similarly, cells containing nitrated tubulin show altered intracellular distribution of the retrograde motor protein, dynein (Eiserich et al., 1999). Further, oxidative stress decreases TrkA levels, and reactive nitrogen species decrease TrkA activation and signaling while increasing levels of p75^NTR^ (Jonnala and Buccafusco, 2001; Olivieri et al., 2002; Ali et al., 2008; Kropf and Fahnestock, 2021). However, whether age-induced nitrative stress selectively alters transport of proNGF via TrkA or p75^NTR^ remains unknown.

This study investigated whether *in vitro* aging and nitrative stress differentially affect retrograde transport of proNGF bound to each of its receptors, TrkA and p75^NTR^, addressed the consequences for signaling and neurodegeneration of the observed receptor-specific alterations, and determined whether age-induced nitrative stress affects TrkA and p75^NTR^ levels as a potential mechanism. These results are important because the biological activity of proNGF depends on its receptors (Masoudi et al., 2009), and receptor specific alterations in proNGF transport and signaling may contribute to neurodegeneration and loss of cognitive function in aging and Alzheimer’s disease.

## 2. Methods

**Figure 1:**
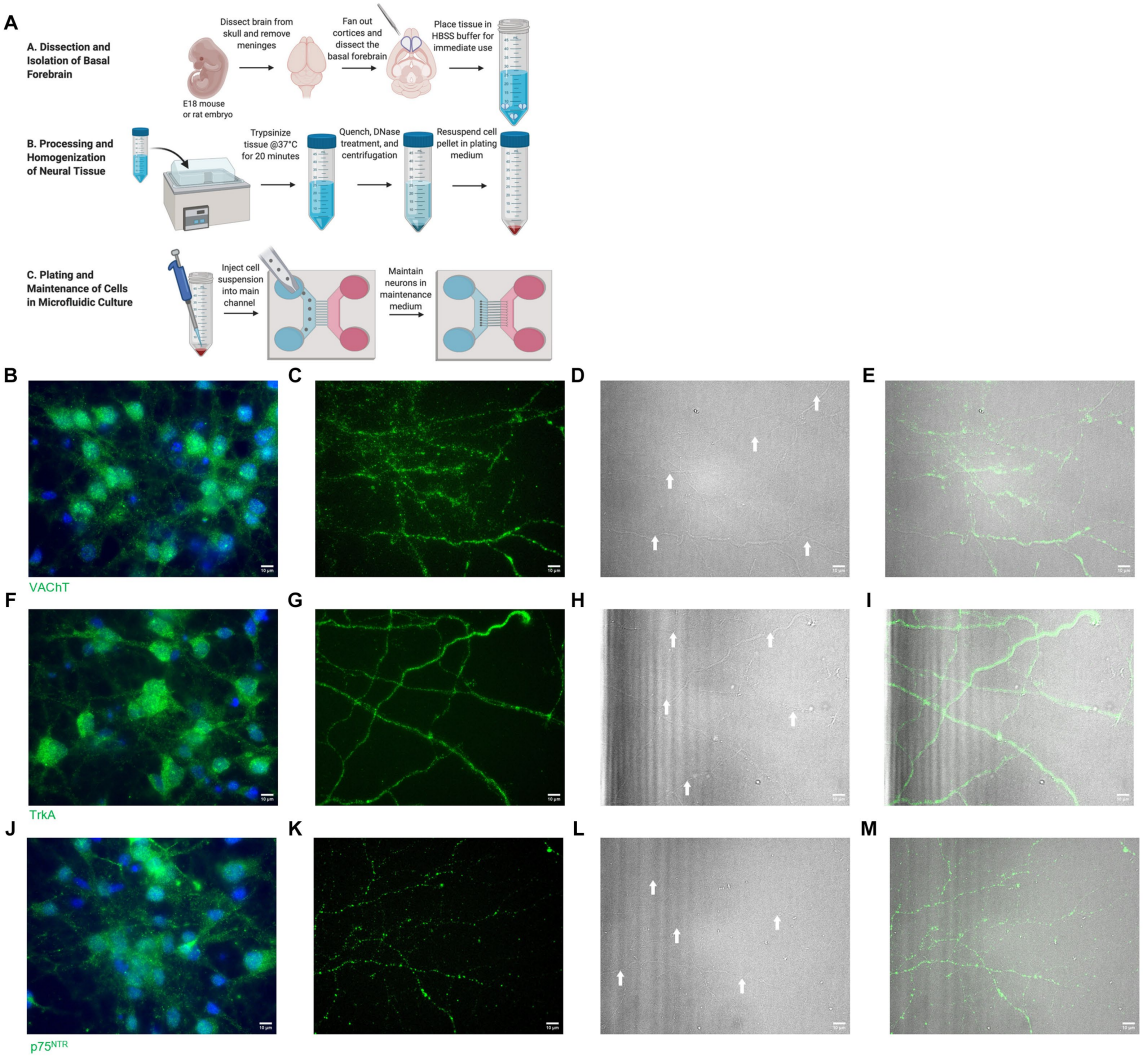
**(A)** Basal forebrain neuron isolation protocol. Reproduced from Shekari and Fahnestock (2022) with permission from Springer. **(B–E)** Isolated basal forebrain neurons stain positive for VAChT (green) at the cell bodies **(B)** and axon terminals **(C)**. Brightfield images of the axon terminals **(D)** show robust arborization of axon terminals. White arrows identify axons. DAPI (blue) stains nuclei **(B)**. **(E)** Overlay of VAChT and brightfield images. **(F–I)** Isolated basal forebrain neurons stain positive for TrkA (green) at the cell bodies **(F)** and axon terminals **(G)**. Brightfield images of the axon terminals **(H)** show robust arborization of axon terminals. White arrows identify axons. DAPI (blue) stains nuclei **(F)**. **(I)** Overlay of TrkA and brightfield images. **(J–M)** Isolated basal forebrain neurons stain positive for p75^NTR^ (green) at the cell bodies **(J)** and axon terminals **(K)**. Brightfield images of the axon terminals **(L)** show robust arborization of axon terminals. White arrows identify axons. DAPI (blue) stains nuclei **(J)**. **(M)** Overlay of p75^NTR^ and brightfield images. Scale bars: 10 μm.

Microfluidic chambers were used to fluidically isolate BFCN cell bodies from their axon terminals, allowing *in vitro* analysis of proNGF axonal transport (Taylor et al., 2005; Shekari and Fahnestock, 2019, 2022). The cholinergic phenotype of these neurons has been previously confirmed by our lab using immunocytochemistry for vesicular acetyl choline transporter (VaChT) and TrkA and was confirmed again in the present study (Figure 1; Shekari and Fahnestock, 2019). BFCN cell culture, proNGF production, axonal transport analysis, and immunostaining were carried out as described in Shekari and Fahnestock (2022) and are reviewed briefly below.

### 2.1. Culturing BFCNs in microfluidic chambers

Microfluidic chambers (Xona Microfluidics, Temecula, California) were prepared following manufacturer’s instructions. Briefly, each chamber was sterilized by addition of 95% ethanol to each well, followed by two washes with phosphate buffered saline (PBS). The wells were then incubated with poly-L-lysine (PLL; Sigma Aldrich, Burlington, Canada) overnight at 37°C, 5% CO_2_, followed by two PBS washes. Finally, the chambers were incubated in plating medium [Neurobasal, 1% Penicillin–Streptomycin, 1X B27 supplement, 1X GlutaMAX supplement, 1% Fetal Bovine Serum, 50 ng/mL BDNF (Peprotech, Rocky Hill, New Jersey), 50 ng/mL NGF (generously provided by Dr. Michael Coughlin, McMaster University, Hamilton, Ontario)] at 37°C, 5% CO_2_ during the dissection.

This study was reviewed and approved by the Animal Research Ethics Board of McMaster University. Basal forebrains were collected from embryonic day 18 (E18) Sprague–Dawley rat, C57BL/6 (wildtype) mouse, or p75^NTR^ exon III knockout mouse (Lee et al., 1992; Walsh et al., 1999) embryos. The tissue was pooled on ice in Hank’s Balanced Salt Solution (HBSS) with 1% penicillin–streptomycin (P/S) and washed five times with HBSS P/S. The tissue was enzymatically digested using 10X trypsin (ThermoFisher, Burlington, Ontario) diluted to 1X in HBSS for 20 min at 37°C with shaking every 5 min. DNAse I (Sigma) was added to 0.1 mg/mL immediately prior to mechanical digestion via trituration with a sterile, fire polished glass pipette. Immediately following trituration, the trypsin was quenched by adding 1 mL of plating medium. The digested tissue was pelleted by centrifugation at 448 x g for 4 min and resuspended in plating medium to a final cell density of 1×10^4^ cells/μl. The plating medium was removed from the chambers, and 20 μL of cells were added directly into the somal channel of the chamber (10 μL in each well). The cells were allowed to adhere for 5 min at 37°C, 5% CO_2_ before the wells were filled with 150 μL of plating medium and left to incubate overnight at 37°C, 5% CO_2_. The next day, the plating medium was replaced with serum-free maintenance medium (Neurobasal, 1% Penicillin–Streptomycin, 1X B27 supplement, 1X GlutaMAX supplement, 50 ng/mL NGF, 50 ng/mL BDNF), which was changed every 48–72 h. The BFCN isolation, processing, and plating process is summarized in Figure 1A.

### 2.2. BFCN aging and confirmation of aged phenotype

BFCNs were maintained for 7–22 days *in vitro* (DIV), as we previously described (Shekari and Fahnestock, 2019, 2022). Young BFCNs (DIV7-9) were analyzed when the axons had extended across the microgrooves. BFCNs were considered aged by DIV18-22. The aged phenotype was confirmed by staining for senescence-associated β-galactosidase, an indicator of cellular aging, using the Senescence β-Galactosidase staining kit (Cell Signaling, Danvers, Massachusetts) and following manufacturer’s instructions. Increased senescence-associated β-galactosidase has been previously reported in BFCNs aged *in vitro* (Shekari and Fahnestock, 2019) and in neurons aged *in vivo* (Geng et al., 2010).

### 2.3. ProNGF axonal transport analysis

#### 2.3.1. ProNGF production

ProNGF receptor specificity was studied using plasmids coding for biotin-accepting, His-tagged mutated versions of cleavage-resistant proNGF [proNGF (R-1G)], which only bind to TrkA (proNGF-KKE) or p75^NTR^ (proNGF-Δ9-13; Hughes et al., 2001; Mahapatra et al., 2009; Sung et al., 2011). ProNGF [R-1G] has an arginine to glycine substitution at the −1 position, a biotin-accepting AVI region, and a 6xHis tag (Sung et al., 2011) and retains full biological activity (Fahnestock et al., 2004b). The KKE mutant contains mutations at Lys^32^, Lys^34^, and Glu^35^, preventing it from binding to p75^NTR^ (Mahapatra et al., 2009). The Δ9-13 mutant has a deletion of amino acids 9 to 13, rendering it unable to bind to TrkA (Hughes et al., 2001). The R-1G mutation was introduced into the mutant proNGF plasmids using the QuikChange II Site-Directed Mutagenesis Kit (Agilent, Santa Clara, California). The mutant proNGF proteins and wildtype proNGF-Avi-His were expressed and purified as described below. The purified biotinylated KKE and Δ9-13 proteins containing the [R-1G] mutation are hereafter referred to as proNGF-KKE and proNGF-Δ9-13, respectively.

HEK293FT cells were grown to 70% confluency in DMEM, 10% fetal bovine serum, 1% Penicillin–Streptomycin, 200 mM GlutaMAX supplement, 50 μM D-biotin (Sigma Aldrich) and 100 mM sodium pyruvate (Sigma Aldrich). The day before transfection, medium was replaced with a serum-free version of the above. The cells were co-transfected with 16 μg of the proNGF, proNGF-KKE, or proNGF-Δ9-13 construct in pcDNA3.1 and an equal amount of a plasmid containing BirA, a biotin ligase (Sung et al., 2011), using TurboFect (ThermoFisher). The transfected cells were incubated for 72 h at 37°C, 5% CO_2_. The medium was removed and incubated with nickel nitrilotriacetic acid (Ni-NTA) resin (ThermoFisher; 40 μL/mL of media) at 4°C for 24 h. The proNGF-Ni-NTA mixture was then added to a reusable nickel purification column (60 mm diameter; BioRad, Mississauga Ontario) for purification of the proNGF-biotin protein, as previously described (Shekari and Fahnestock, 2022). The eluted protein was concentrated by centrifugal filtration in 10 kDa filters (Sigma) at 300 x g, 4°C. The resulting protein was aliquoted and stored at −80°C and its concentration was determined by enzyme-linked immunosorbent assay (ELISA) (Fahnestock et al., 2004b).

#### 2.3.2. Labeling proNGF

Quantum dot 625-streptavidin conjugate (ThermoFisher) was added to 200pM proNGF, proNGF-KKE, or proNGF-Δ9-13 in a 1:1 molar ratio and incubated on ice in the dark for 1 h. The quantum dot (QD) labeled protein was then diluted to 50pM in neurotrophin-free maintenance medium containing Tubulin Tracker Deep Red (ThermoFisher) at a 1:1000X dilution.

#### 2.3.3. Live imaging

Fluorescence microscopy was used to analyze proNGF-QD accumulation at the proximal axons and cell bodies following its addition to BFCN axon terminals only. 80 μL of the proNGF-QD solution described above was added directly into the axon channel of the chambers. Neurotrophin-free maintenance medium containing Tubulin Tracker Deep Red (1:1000) and 2 drops/ml NucBlue Live ReadyProbes Reagent (ThermoFisher) were added to the cell body wells of the chambers. BFCNs were incubated for 1 h at 37°C, 5% CO_2_, followed by three washes with neurotrophin-free maintenance medium, maintaining the volume difference between the cell body and axon wells. The chambers were then imaged using the 60x objective of an EVOS2 FL epifluorescence microscope with an environmental chamber at 37°C, 5% CO_2_. Qdot625 and Cy5 filters were set at light intensity 0.01. All images were analyzed using ImageJ. Quantum dot fluorescence in the axon terminal channel was normalized to tubulin fluorescence (QD/tubulin fluorescence). Since proNGF is rapidly and efficiently taken up and transported by BFCNs, individual particles cannot be tracked for generation of kymographs or quantification of speed and pause duration (Shekari and Fahnestock, 2019). As previously reported, it is extremely difficult to distinguish the transport pathways of each proNGF-QD particle due to the efficient uptake and transport of proNGF-QD along the length of the microgrooves (Shekari and Fahnestock, 2019). However, because individual quantum dots were distinguishable at the proximal axons at the cell body end of the microgrooves, proNGF accumulation was quantified in this compartment by normalizing the number of quantum dots to tubulin fluorescence (#QDs/tubulin fluorescence).

### 2.4. Neurodegeneration analysis

Tubulin images from axonal transport assays were analyzed for neurodegeneration as previously described (Kneynsberg et al., 2016). Briefly, tubulin images were thresholded using the Otsu plugin on ImageJ. Axon fragmentation was analyzed by measuring particle size using the “analyze particle” function on ImageJ with size set from 0-infinity and circularity set from 0 to 1. The number of particles was normalized to the total particle area.

### 2.5. Detection and inhibition of nitric oxide

Nitric Oxide (NO) was detected using 4-Amino-5-Methylamino-2′,7’-Difluorofluorescein Diacetate (DAF-FM, ThermoFisher) following manufacturer’s instructions. Briefly, BFCNs were rinsed once with maintenance medium made without phenol red (hereafter referred to as colorless maintenance medium) prior to staining. BFCNs were incubated in colorless maintenance medium containing 5 μM DAF-FM and 2 drops/ml NucBlue Live ReadyProbes Reagent (ThermoFisher) at 37°C, 5% CO_2_ for 1 h. All wells were washed three times with colorless maintenance medium, followed by a 15-min incubation at 37°C, 5% CO_2_. BFCNs were placed in the environmental chamber of the EVOS2 FL microscope at 37°C, 5% CO_2_. The imaging location was identified based on DAPI staining. The DAF-FM fluorescence (detected using a YFP filter, light intensity 0.01) in the entire image was normalized to the total number of cells in the image using ImageJ.

Nitric oxide synthase was inhibited using N(G)-Nitro-L-arginine methyl ester (L-NAME, Sigma). 1 mM L-NAME in maintenance medium was added to all wells of the BFCN chambers for 24 h at 37°C, 5% CO_2_. Axonal transport of proNGF-KKE and proNGF-Δ9-13 was then analyzed as described above using neurotrophin-free maintenance medium containing either 1 mM L-NAME or an equal volume of vehicle.

### 2.6. Immunocytochemistry

50pM proNGF-Δ9-13, proNGF-KKE, or heat-inactivated wildtype proNGF (negative control) in neurotrophin-free maintenance medium was added to the axonal compartment of the chambers and incubated at 37°C, 5% CO_2_ for 15 min. BFCNs were washed once with PBS and fixed in 4% paraformaldehyde at room temperature for 30 min. Each well was rinsed once with PBS prior to permeabilization with 0.2% Triton-X100 in PBS at room temperature for 30 min. BFCNs were blocked in 3% bovine serum albumin, 5% fetal bovine serum, 1% penicillin–streptomycin in PBS at room temperature for 30 min. Primary antibodies were added at the following dilutions and incubated overnight at 4°C: anti-phospho p44/42 MAPK (ERK1/2) Thr 202/Tyr 204 rabbit polyclonal antibody (1:250, Cell Signaling), anti p44/42 MAPK (ERK1/2) mouse monoclonal antibody (1:100, Cell Signaling), anti-phospho SAPK/JNK Thr 183/Tyr 185 mouse monoclonal antibody (1:200, Cell Signaling), anti-JNK1/2/3 rabbit polyclonal antibody (1:100, Origene, Rockville, Maryland), anti-VAChT rabbit polyclonal antibody (1:500, Santa Cruz Biotechnology), anti-TrkA rabbit polyclonal antibody (1:200, Sigma), and anti-p75^NTR^ rabbit polyclonal antibody (1:500, Abcam). BFCNs were washed with blocking solution 3 times prior to a 2-h incubation with secondary antibodies, Alexa Fluor goat anti-rabbit 488 (ThermoFisher) and Alexa Fluor goat anti-mouse 647 (ThermoFisher), each diluted 1:1000 in blocking solution. Secondary antibodies were replaced with PBS including 2 drops/ml NucBlue Fixed Cell ReadyProbes Reagent (ThermoFisher) for 10 min, followed by five PBS rinses. BFCNs were then imaged with an EVOS2FL microscope using the YFP (light intensity 0.01 for phospho-ERK and JNK, 0.007 for TrkA and p75^NTR^), Cy5 (light intensity 0.01), and DAPI (light intensity 0.0002) filters. 30 image fields were averaged per group, taken from 10 images per chamber in three independent experiments.

For analysis of ERK and JNK signaling, the mean fluorescence values of the entire field of each channel were analyzed using ImageJ. pERK values were normalized to total ERK, and pJNK values were normalized to total JNK.

ImageJ was used for analysis of TrkA and p75^NTR^ levels following instructions provided by the imaging specialists at McMaster’s Center for Advanced Light Microscopy. Briefly, any background signal was first subtracted using the “Subtract Background” function (rolling ball radius 100 pixels, sliding paraboloid), followed by application of the “Gaussian Blur” filter (Sigma 2.00). Images were then thresholded using the “Huang” threshold setting. The resulting mask was then applied to the original image using the “Image Calculator” function (Image 1: mask, Image 2: original image, operation: multiply, 32-bit result). The mean fluorescence was then measured from the resulting output with the threshold limited to 1. This method of analysis measures the mean fluorescence only from areas of the image that contain positive signal. The mean fluorescence value accounts for the number of pixels within the measured area. Images containing a greater number or size of cells or axons contain larger areas of positive staining and more pixels. Therefore, any differences in cell or axon number between groups are accounted for using this method.

### 2.7. Western blotting

BFCNs were isolated as described in Section 2.1 above and plated in 6-well plates at a density of 10^6^ cells/well. Cells were rinsed once with PBS prior to lysing with radioimmunoprecipitation assay (RIPA) buffer containing Halt protease inhibitor cocktail (ThermoFisher) and Roche PhosSTOP phosphatase inhibitors (Sigma). Pheochromocytoma (PC12) cell lines PC12nnr5 (Green et al., 1986) and PC12nnr5B5 (Baskey et al., 2002) were grown in RPMI, 10% Horse Serum (ThermoFisher), 5% Fetal Bovine Serum (ThermoFisher), 1% Penicillin–Streptomycin (Sigma). Following 7 days at 37°C, 5% CO_2_, cells were lysed in RIPA buffer containing Halt protease inhibitor cocktail and Roche PhosSTOP phosphatase inhibitors. Cell lysates were collected by centrifugation at 13250 x g for 5 min. The supernatant was collected and stored at −80°C. Hippocampal tissue was dissected from adult C57BL/6 N (wildtype) or p75^NTR^ exon III knockout mice (Lee et al., 1992; Walsh et al., 1999), flash frozen in liquid nitrogen, and stored at −80°C prior to homogenization by sonication in RIPA buffer containing Halt protease inhibitor cocktail and Roche PhosSTOP phosphatase inhibitors. Tissue lysates were collected by centrifugation at 12000 x g for 15 min at 4°C. Protein concentrations of all lysates were determined by DC Protein Assay (BioRad). Protein lysates from one to three independent experiments were run on three 12% sodium dodecyl-sulfate polyacrylamide gel electrophoresis (SDS-PAGE) gels and electrophoretically transferred onto polyvinylidene fluoride (PVDF) membranes (Millipore). Membranes were dipped in 100% methanol and air dried, then incubated in 5% skim milk in 20 mM Tris-buffered saline pH7.6–0.1% Tween 20 detergent (TBS-T) with primary antibody. Rat monoclonal anti-p75 NGF Receptor (Abcam; dilution 1:500) was incubated 15 h at 4°C, mouse monoclonal anti-β-Actin (Sigma-Aldrich; dilution 1:10000) was incubated for 45 min at room temperature, and rabbit polyclonal anti-TrkA (Cell Signaling Technologies; 1:500) was incubated for 2 h at room temperature and then 15 h at 4°C. Following three five-minute washes in TBS-T, horseradish peroxidase conjugated anti-rabbit (ThermoFisher; 1:5000), anti-mouse (Cell Signaling Technologies; 1:2000), or anti-rat (Abcam; 1:1000) secondary antibodies in 5% skim milk in TBS-T were incubated for 1 h at room temperature. Following TBS-T washes, Clarity ECL (BioRad) peroxide and luminol/enhancer reagents for anti-β-actin or SuperSignal West Femto (ThermoFisher) peroxide and luminol/enhancer reagents for anti-p75^NTR^ and anti-TrkA were added dropwise onto the PVDF membranes. Image Lab v.6.0.1 (BioRad) was used to quantify band intensities, and each band was normalized to its β-actin loading control.

### 2.8. Statistics

All statistical analyzes were conducted in GraphPad Prism 8.0.2 (Dotmatics, Boston, Massachusetts). For comparison between two groups, unpaired t-tests were used. Differences between three or more groups were analyzed using one-way analysis of variance (ANOVA) followed by Tukey’s multiple comparisons for significant ANOVAs (*p* < 0.05). Outliers were determined using the robust regression and outlier removal (ROUT) method (Q = 1%) (Motulsky and Brown, 2006). Experiments were repeated on three independent occasions using BFCNs from separate litters.

## 3. Results

**Figure 2:**
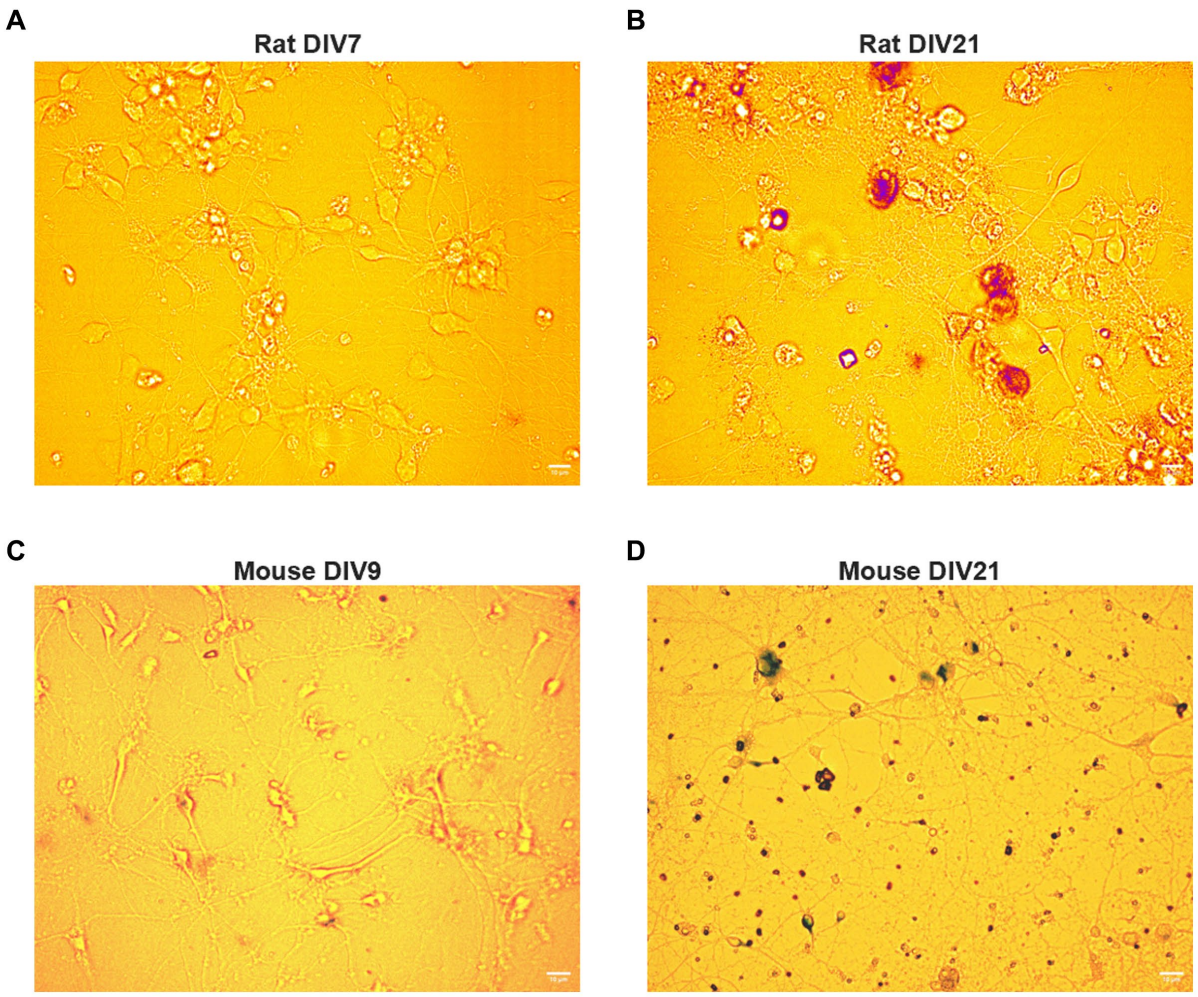
Rat and mouse BFCNs aged *in vitro* stain positive for senescence-associated beta-galactosidase. Aged (DIV17-21) and young (DIV7-10) basal forebrain neurons were stained for senescence-associated beta-galactosidase, a well-validated marker of cellular aging, using a colorimetric staining kit. **(A)** Senescence-associated beta-galactosidase is undetectable in young rat BFCNs. **(B)** Rat basal forebrain neurons aged *in vitro* stain positive for senescence-associated beta-galactosidase (red). DIV: days *in vitro*. Scale bars: 10 μm. **(C)** Senescence-associated beta-galactosidase is undetectable in young mouse BFCNs. **(D)** Mouse basal forebrain neurons aged *in vitro* stain positive for senescence-associated beta-galactosidase (purple). DIV, days *in vitro*. Scale bars: 10 μm.

BFCNs were isolated and processed as described in Materials and Methods (Figure 1A). The cholinergic phenotype of the neurons obtained from this process was confirmed using immunocytochemistry for TrkA, vesicular acetylcholine transporter (VAChT), and p75^NTR^ (Figures 1B–J). BFCNs were aged *in vitro* for 7–22 days. The aged phenotype has been previously validated by our lab (Shekari and Fahnestock, 2019) and was confirmed again in the present study by staining for senescence-associated β-galactosidase, a well-established marker of cellular aging (Dimri et al., 1995; Uday Bhanu et al., 2010). Increased accumulation of this marker was observed by 21 days *in vitro* (DIV) in both rat (Figures 2A,B) and mouse (Figures 2C,D) BFCNs. In transport experiments, BFCN axon terminals, proximal axons, and cell bodies were imaged.

### 3.1. Young BFCNs transport proNGF via TrkA

**Figure 3:**
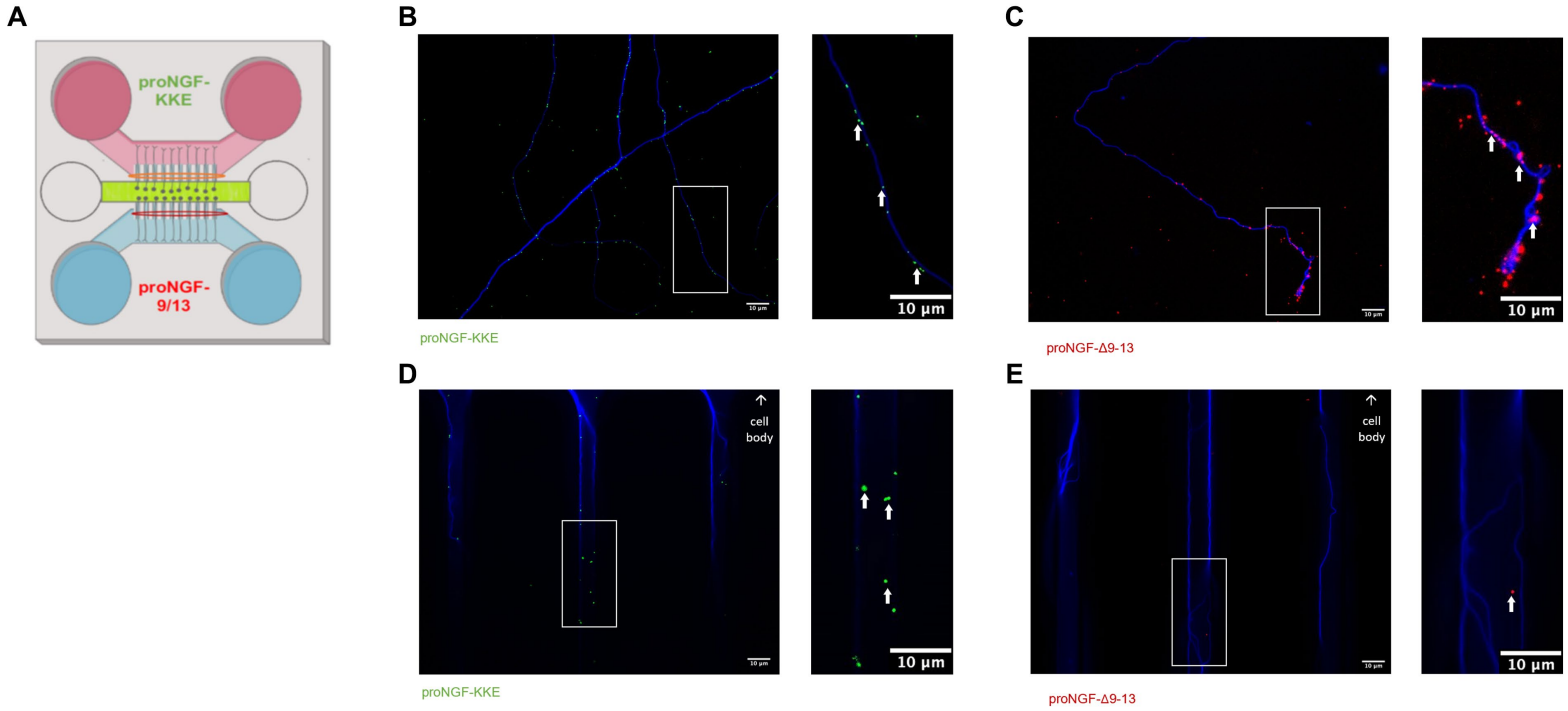
Young BFCNs transport proNGF via TrkA but not via p75^NTR^. Rat basal forebrain cholinergic neurons (BFCNs) were cultured in triple channel devices containing 2 sets of fluidically isolated microgrooves as indicated in panel **A**. White boxes indicate locations of magnified inserts (right panels). White arrows identify quantum dots. BFCNs were co-incubated with 50pM TrkA-binding proNGF-KKE [pink section, panel **(A)**] and p75^NTR^-binding proNGF-Δ9-13 [blue section, panel **(A)**]. Both proteins were found within the axon terminals after 30 min **(B,C)**, demonstrating binding. proNGF-KKE (green dots) was observed within microgrooves proximal to cell bodies after 1 h **(D)**. proNGF-Δ9-13 (red dots) was not observed within microgrooves after 1 h **(E)**. Representative results from 2 chambers per group.

**Figure 4:**
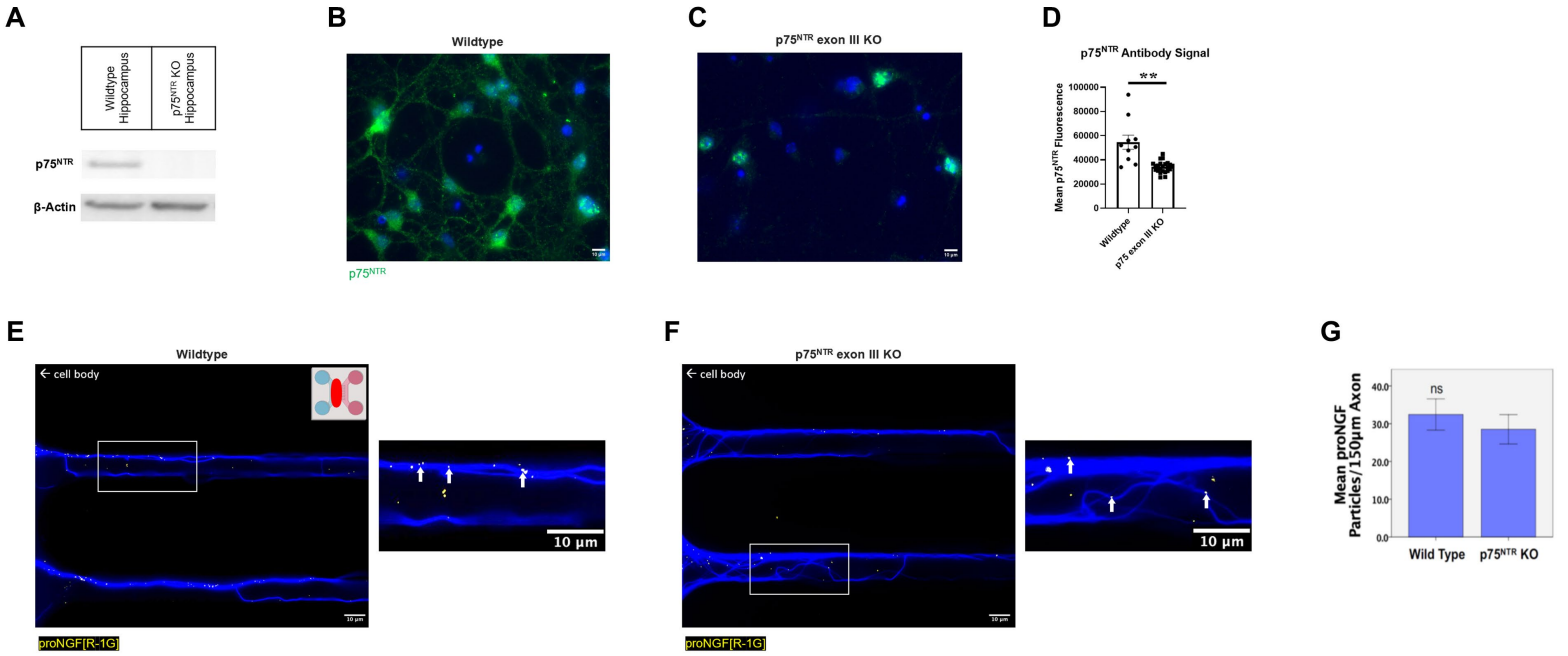
ProNGF is transported in both wildtype and p75^NTR^ exon III knockout mouse BFCNs. **(A)** Hippocampal tissue obtained from p75^NTR^ exon III knockout and C57BL/6 (wildtype) mice was assayed for p75^NTR^ by western blotting. p75^NTR^ was detectable only in the wildtype tissue. β-Actin was used as a loading control. **(B–D)** DIV8-9 mouse p75^NTR^ exon III knockout BFCNs and mouse C57BL/6 (wildtype) BFCNs were stained for p75^NTR^ using immunocytochemistry. DAPI is shown in blue and p75^NTR^ is shown in green. **(B)** Wildtype mouse BFCNs exhibit strong staining for p75^NTR^. **(C)** Some antibody signal is detectable in p75^NTR^ exon III knockout BFCNs. **(D)** Quantification of mean p75^NTR^ antibody signal in wildtype BFCNs compared to p75^NTR^ exon III knockout BFCNs. Error bars represent SEM, *n* = 10 wildtype images and *n* = 23 p75^NTR^ exon III knockout images obtained from 1 chamber per group. **p < 0.01. No outliers were identified using ROUT (Q = 1%). **(E–G)** The retrograde axonal transport of proNGF did not differ between BFCNs cultured from wildtype vs. p75^NTR^ knockout (KO) mice. Wildtype [panel **(E)**] and p75^NTR^ KO neurons [panel **(F)**] were incubated with proNGF-QD (yellow dots) at the axonal compartment only [indicated by the pink section of the schematic inset in panel **(E)**] for 1 h before imaging. The red oval indicates where the images were taken. White boxes indicate locations of magnified inserts (right panels). Arrows indicate quantum dots, which appear white when colocalized with tubulin. Experiments were completed three times. **(G)** Quantification of number of quantum dots per 150 μm axon length. *N* = 60 microgrooves from 3 chambers per group, mean +/− SE. ns (not significant) *p* = 0.46, Student’s t-test. Scale bars: 10 μm.

To determine which receptor transports proNGF in young rat BFCNs, proNGF-KKE and proNGF-Δ9-13 were added to the top and bottom wells, respectively, of a triple microfluidic chamber device containing DIV8-14 BFCNs (Figure 3A). Both proNGF-KKE (Figure 3B) and proNGF-Δ9-13 (Figure 3C) were observed within the axon terminals. However, only proNGF-KKE was transported to the proximal axons (Figure 3D), while proNGF-Δ9-13 was not (Figure 3E). These results suggest that p75^NTR^ is not required for retrograde transport of proNGF in young BFCNs. To confirm these findings, retrograde transport of proNGF capable of binding to both receptors was analyzed in DIV8-14 p75^NTR^ knockout mouse BFCNs. The absence of p75^NTR^ in the knockout BFCNs was confirmed using western blotting (Figure 4A) and immunocytochemistry (Figures 4B–D). The reduced but detectable signal seen with immunostaining in panel C may be due to the exon III truncated form of p75^NTR^ (Lee et al., 1992; Walsh et al., 1999). The antibody used for western blot differs from the antibody used in immunohistochemistry, which is directed against the intracellular domain of p75^NTR^ and therefore recognizes both the full-length and truncated forms (Von Schack et al., 2001; ABCAM, 2023). No differences were observed in proNGF accumulation at the proximal axons in wildtype mouse vs. p75^NTR^ knockout mouse BFCNs (Figures 4E–G; *p* = 0.46). These results indicate that young BFCNs transport proNGF via TrkA.

### 3.2. *In vitro* aging decreases retrograde transport of proNGF via TrkA but increases retrograde transport of proNGF via p75^NTR^

We have previously shown that retrograde transport of proNGF is impaired in BFCNs aged *in vitro* (Shekari and Fahnestock, 2019). To determine whether aging differentially affects axonal transport of proNGF via each of its receptors, we analyzed retrograde transport of proNGF-KKE and proNGF-Δ9-13 in young (DIV7-9) and aged rat BFCNs (DIV20-22).

**Figure 5:**
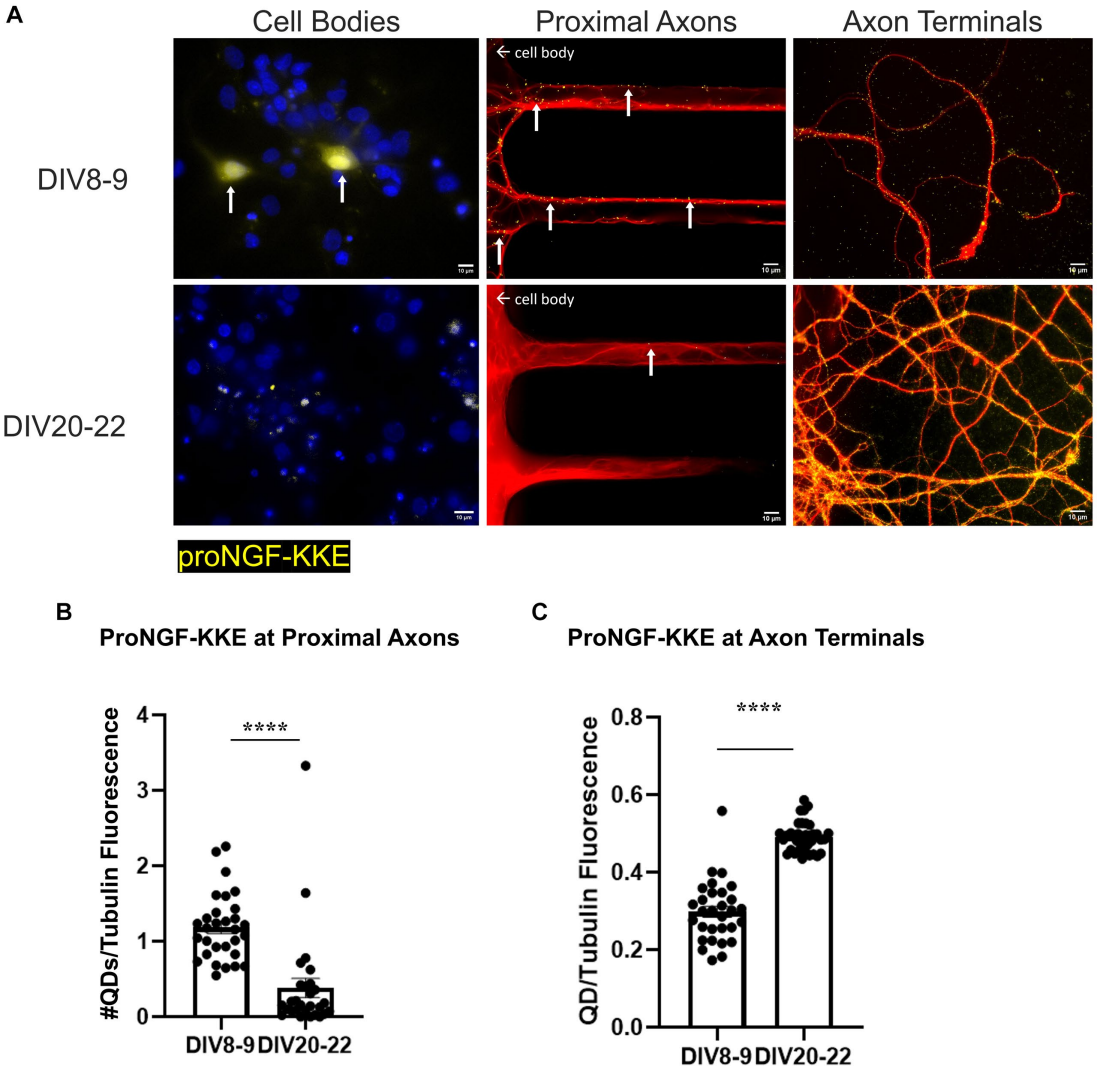
Retrograde transport of proNGF-KKE is impaired with *in vitro* age in BFCNs. 50pM of quantum dot labeled proNGF-KKE was added to the axon terminals of young (DIV8-9) or aged (DIV20-22) rat BFCNs for 1 h prior to analysis of quantum dot accumulation at BFCN axon terminals, proximal axons, and cell bodies. proNGF-KKE is shown in yellow, tubulin is shown in red, and DAPI is shown in blue. **(A)** ProNGF-KKE was observed in young but not aged BFCN cell bodies. White arrows indicate quantum dots that accumulated in the proximal axons and cell bodies. Aged BFCNs exhibited decreased accumulation of proNGF-KKE at the proximal axons and increased accumulation of proNGF-KKE at the axon terminals compared to young BFCNs. **(B,C)** Quantification of the images shown in **A**. **(B)** Proximal axons: young *n* = 30 images, aged *n* = 28 images. The highest two points in the DIV20-22 group were detected as outliers using ROUT (Q = 1%). Removal of these outliers did not change the observed value of *p*. **(C)** Axon terminals: young *n* = 30 images, aged *n* = 37 images. No outliers were detected using ROUT (Q = 1%). All sample sizes are taken from three separate chambers in three independent experiments. Error bars represent SEM. ****p < 0.0001. DIV, days *in vitro*; QD, quantum dot. Scale bars: 10 μm.

ProNGF-KKE accumulation was observed in young but not aged rat BFCN cell bodies (Figure 5A). *In vitro* aging decreased retrograde transport of proNGF-KKE, demonstrated by decreased accumulation of proNGF-KKE at the proximal axons (Figures 5A,B; *p* < 0.0001) and increased accumulation of proNGF-KKE at the axon terminals (Figures 5A,C; *p* < 0.0001) in aged BFCNs compared to young BFCNs.

**Figure 6:**
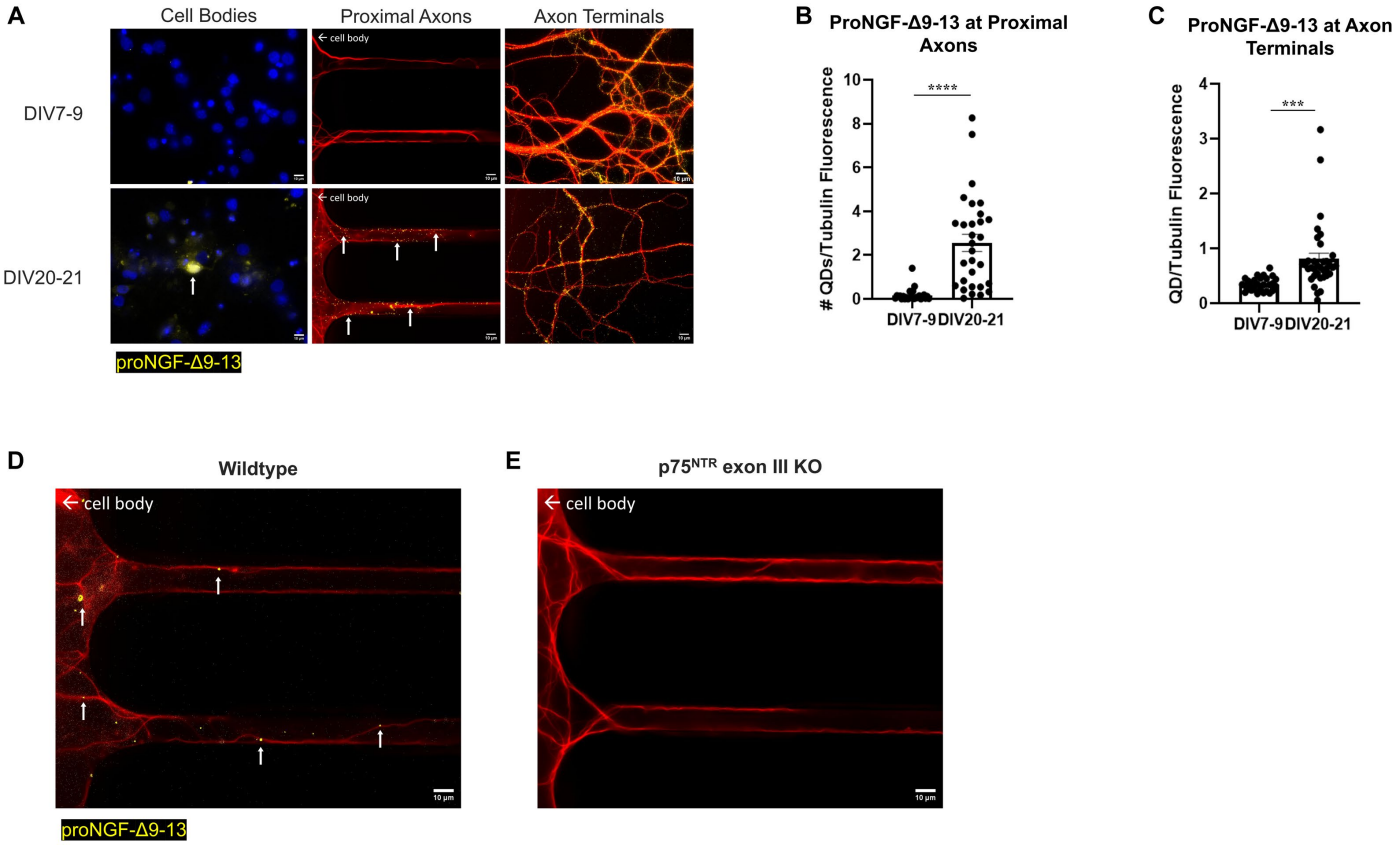
Retrograde transport of proNGF-Δ9-13 is increased with *in vitro* age in BFCNs. 50pM of quantum dot labeled proNGF-Δ9-13 was added to the axon terminals of young (DIV7-9) or aged (DIV20-21) rat BFCNs for 1 h prior to analysis of quantum dot accumulation at BFCN axon terminals, proximal axons, and cell bodies. proNGF-Δ9-13 is shown in yellow, tubulin is shown in red, and DAPI is shown in blue. White arrows indicate quantum dots that accumulated in the proximal axons and cell bodies. **(A)** ProNGF-Δ9-13 was observed in aged but not young BFCN cell bodies (white arrows). Aged BFCNs exhibited increased accumulation of proNGF-Δ9-13 at the proximal axons and increased accumulation of proNGF-Δ9-13 at the axon terminals relative to young BFCNs. **(B,C)** Quantification of the images shown in **A**. **(B)** At the proximal axons: *n* = 27 images for the young BFCNs, and *n* = 30 images for the aged BFCNs. The highest five points in the DIV7-9 group were detected as outliers using ROUT (Q = 1%), and no outliers were detected in the DIV20-21 group. Removal of these outliers did not affect the observed value of *p*. **(C)** At the axon terminals: *n* = 28 images for the young BFCNs, and *n* = 35 images for the aged BFCNs. The highest two points in the DIV20-21 group were detected as outliers using ROUT (Q = 1%). Removal of these outliers changed the value of p from *p* = 0.0003 (***) to *p* < 0.0001 (****). All sample sizes are taken from three separate chambers in three independent experiments. ***p < 0.001, ****p < 0.0001. DIV, days *in vitro*; QD, quantum dot. **(D,E)** 50pM of quantum dot labeled proNGF-Δ9-13 was added to the axon terminals of aged (DIV21) wildtype (C57BL/6) mouse BFCNs **(D)** or p75^NTR^ exon III knockout BFCNs **(E)** for 1 h prior to imaging BFCN proximal axons. **(D)** proNGF-Δ9-13 accumulates in wildtype proximal axons. **(E)** proNGF-Δ9-13 is not observed in p75^NTR^ exon III knockout proximal axons. Scale bars: 10 μm.

Conversely, *in vitro* aging increased retrograde transport of proNGF-Δ9-13. Rat BFCNs aged *in vitro* had quantum dot accumulation in the cell bodies and an increased number of proNGF-Δ9-13 quantum dots accumulating at the proximal axons (Figures 6A,B; *p* < 0.0001) as well as increased levels of proNGF-Δ9-13 at the axon terminals (Figures 6A,C; *p* = 0.0003) compared to young BFCNs.

Accumulation of proNGF-Δ9-13 was also observed in aged wildtype mouse BFCN proximal axons (Figure 6D) but not in aged p75^NTR^ exon III knockout BFCN proximal axons (Figure 6E), indicating that the transport of proNGF-Δ9-13 observed in aged BFCNs is via p75^NTR^. Together, these results indicate that retrograde transport of proNGF-KKE is impaired with *in vitro* age, while retrograde transport of proNGF-Δ9-13 is enhanced in aged BFCNs. This suggests that aging decreases retrograde transport of proNGF bound to TrkA while increasing retrograde transport of proNGF bound to p75^NTR^.

### 3.3. ProNGF-Δ9-13 induces greater apoptotic signaling and less neurotrophic signaling than proNGF-KKE

To determine whether the observed receptor specific proNGF transport changes are functionally significant, either proNGF-KKE or proNGF-Δ9-13 was added to rat BFCN axon terminals for 15 min prior to analysis of signaling factor activity at the cell bodies using immunocytochemistry. As a negative control, proNGF was inactivated by boiling for 5 min at 100°C. To confirm inactivation, either boiled proNGF or vehicle was applied to rat BFCN axon terminals for 15 min prior to analysis of signaling factor activation at the cell bodies. The boiled proNGF did not activate the pro-apoptotic signaling factor, JNK (Supplementary Figure S1A; *p*  =  0.96), or the neurotrophic signaling factor, ERK (Supplementary Figure S1B; *p* = 0.99), compared to vehicle, indicating full inactivation. ProNGF-Δ9-13 and proNGF-KKE were included as positive controls for activation of JNK and ERK, respectively. ProNGF-Δ9-13 induced greater JNK activation than both inactivated proNGF (*p* < 0.0001) and vehicle (*p* < 0.0001; Supplementary Figure S1A). ProNGF-KKE induced greater ERK activation than both inactivated proNGF (*p* = 0.026) and vehicle (*p* = 0.027; Supplementary Figure S1B).

**Figure 7:**
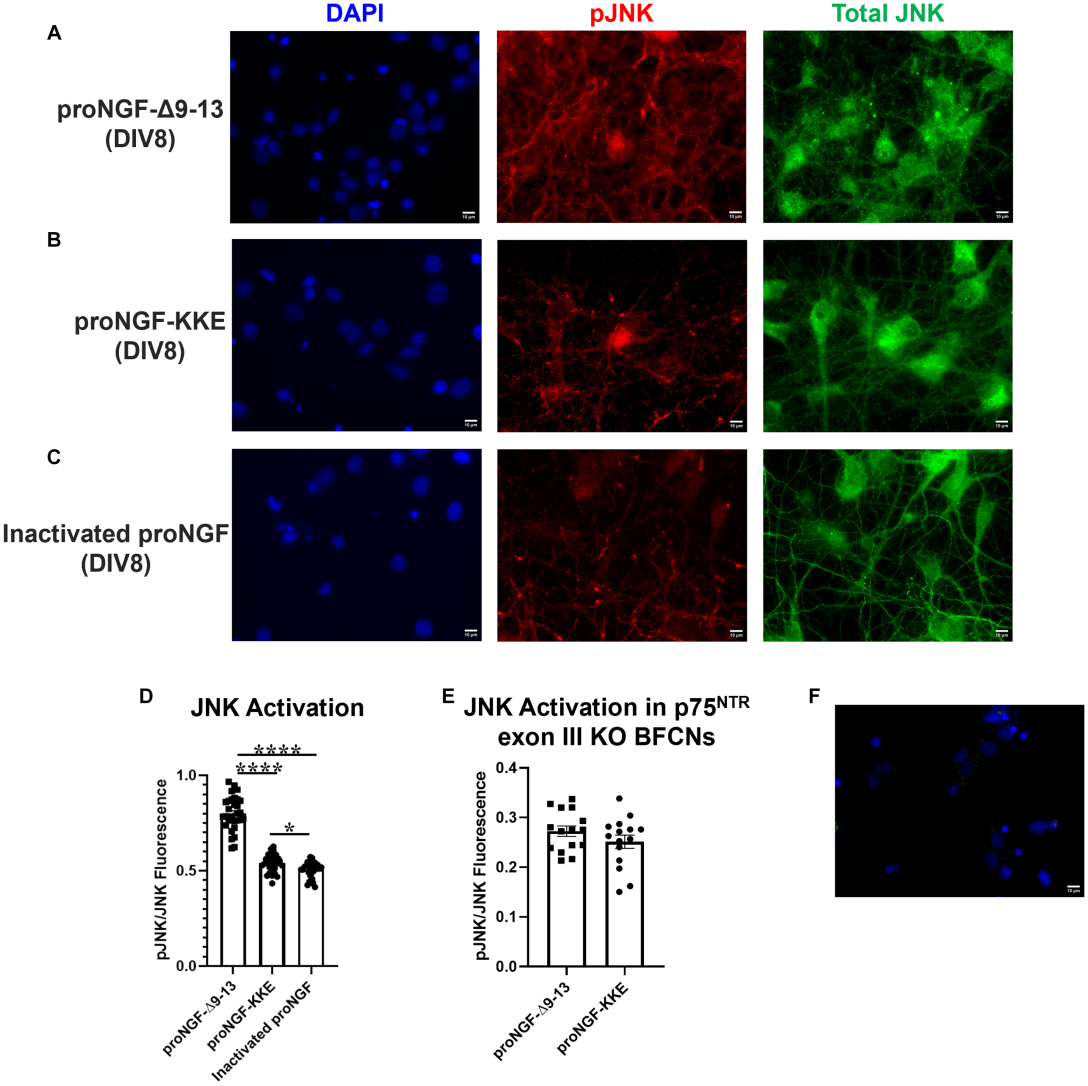
ProNGF-Δ9-13 at axon terminals induces greater apoptotic signaling at cell bodies than proNGF-KKE. ProNGF-Δ9-13, proNGF-KKE, or inactivated proNGF were applied to rat BFCN axon terminals for 15 min, followed by quantification of signaling factor activation at BFCN cell bodies. ProNGF-Δ9-13 **(A)** caused greater activation (phosphorylation) of the pro-apoptotic signaling factor, JNK, relative to proNGF-KKE **(B)** and inactivated proNGF **(C)**. pJNK is shown in red, total JNK in green, and DAPI in blue. DIV, days *in vitro*. **(D)** Quantification of the images shown in **A–C**. Error bars represent SEM, *n* = 30 images/group taken from three chambers in three independent experiments. One-way ANOVA and *post hoc* Tukey test, *p < 0.05, ****p < 0.0001. **(E)** ProNGF-Δ9-13 or proNGF-KKE were applied to mouse p75^NTR^ exon III knockout BFCN axon terminals for 15 min, followed by quantification of JNK activation at BFCN cell bodies. Activation of the pro-apoptotic signaling factor, JNK, induced by proNGF-Δ9-13 was not different from that induced by proNGF-KKE, Student’s *t*-test, *p* > 0.05. Error bars represent SEM, *n* = 15 images/group taken from one chamber. **(F)** Immunocytochemistry was conducted on DIV8 rat BFCNs as described in Materials and Methods, with the elimination of primary antibodies. Minimal fluorescent signal was detected for each secondary antibody under these conditions. Alexa Fluor goat anti-rabbit 488 shown in green, Alexa Fluor goat anti-mouse 647 shown in red, DAPI shown in blue. Scale bars: 10 μm.

Axonal incubation with proNGF-Δ9-13 resulted in increased activation of JNK in the cell bodies relative to proNGF-KKE (Figures 7A,B,D; *p* < 0.0001) and to inactivated proNGF (Figures 7A,C,D; *p* < 0.0001). To confirm that the activation of JNK caused by proNGF-Δ9-13 was caused by activation of p75^NTR^, JNK activation was analyzed in p75^NTR^ exon III knockout mouse BFCNs following incubation with either proNGF-Δ9-13 or proNGF-KKE. Differences in JNK activation induced by proNGF-Δ9-13 compared to proNGF-KKE were abolished in p75^NTR^ knockout BFCNs (*p* = 0.221, Figure 7E). To confirm the specificity of the fluorescent signal, secondary antibodies were incubated without primary antibodies in DIV8 BFCNs. Minimal signal was detected in the absence of the primary antibodies (Figure 7F).

**Figure 8:**
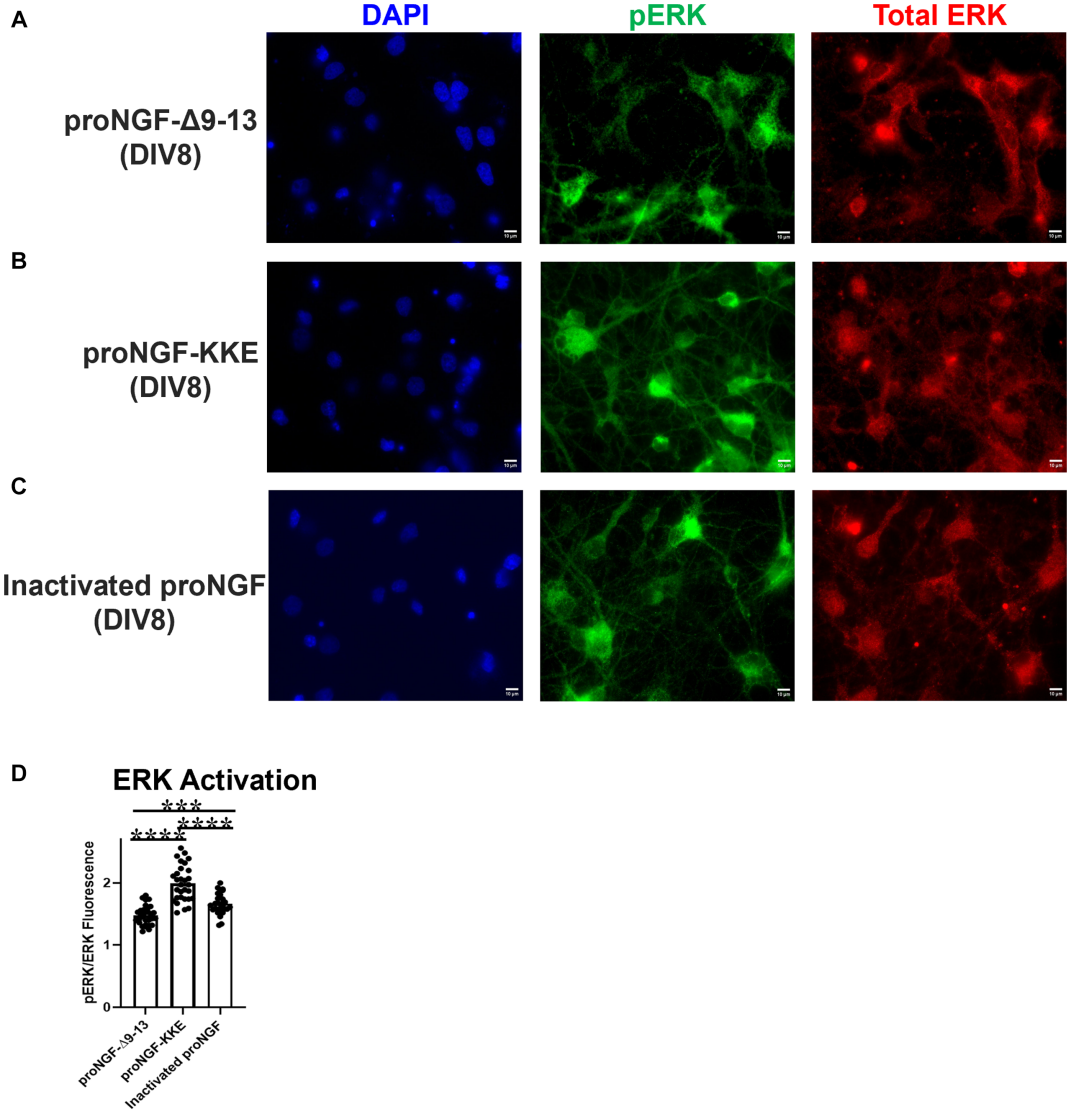
ProNGF-KKE at axon terminals induces greater neurotrophic activity at cell bodies than proNGF-Δ9-13. ProNGF-Δ9-13, proNGF-KKE, or inactivated proNGF were applied to rat BFCN axon terminals for 15 min, followed by quantification of signaling factor activation at BFCN cell bodies. ProNGF-KKE **(B)** caused greater activation (phosphorylation) of the neurotrophic signaling factor, ERK, compared to proNGF-Δ9-13 **(A)** and inactivated proNGF **(C)**. pERK is shown in green, total ERK in red, and DAPI in blue. DIV: days *in vitro*. **(D)** Quantification of the images shown in **A–C**. Error bars represent SEM. *N* = 30 images/group taken from three chambers in three independent experiments. ***p < 0.001, ****p < 0.0001. Scale bars: 10 μm.

The activity of ERK induced by proNGF-KKE was greater than that induced by proNGF-Δ9-13 (Figures 8A,B,D; *p* < 0.0001) and by inactivated proNGF (Figures 8B–D; *p* < 0.0001). Interestingly, proNGF-Δ9-13 induced less ERK activation than inactivated proNGF (Figures 8A,C,D; *p* = 0.0002), indicating that this construct inhibits some baseline ERK activity. Together, these results indicate that axonal proNGF induces neurotrophic signaling in the cell bodies via TrkA but apoptotic signaling in the cell bodies via p75^NTR^.

### 3.4. ProNGF-Δ9-13 causes greater neurodegeneration than proNGF-KKE

**Figure 9:**
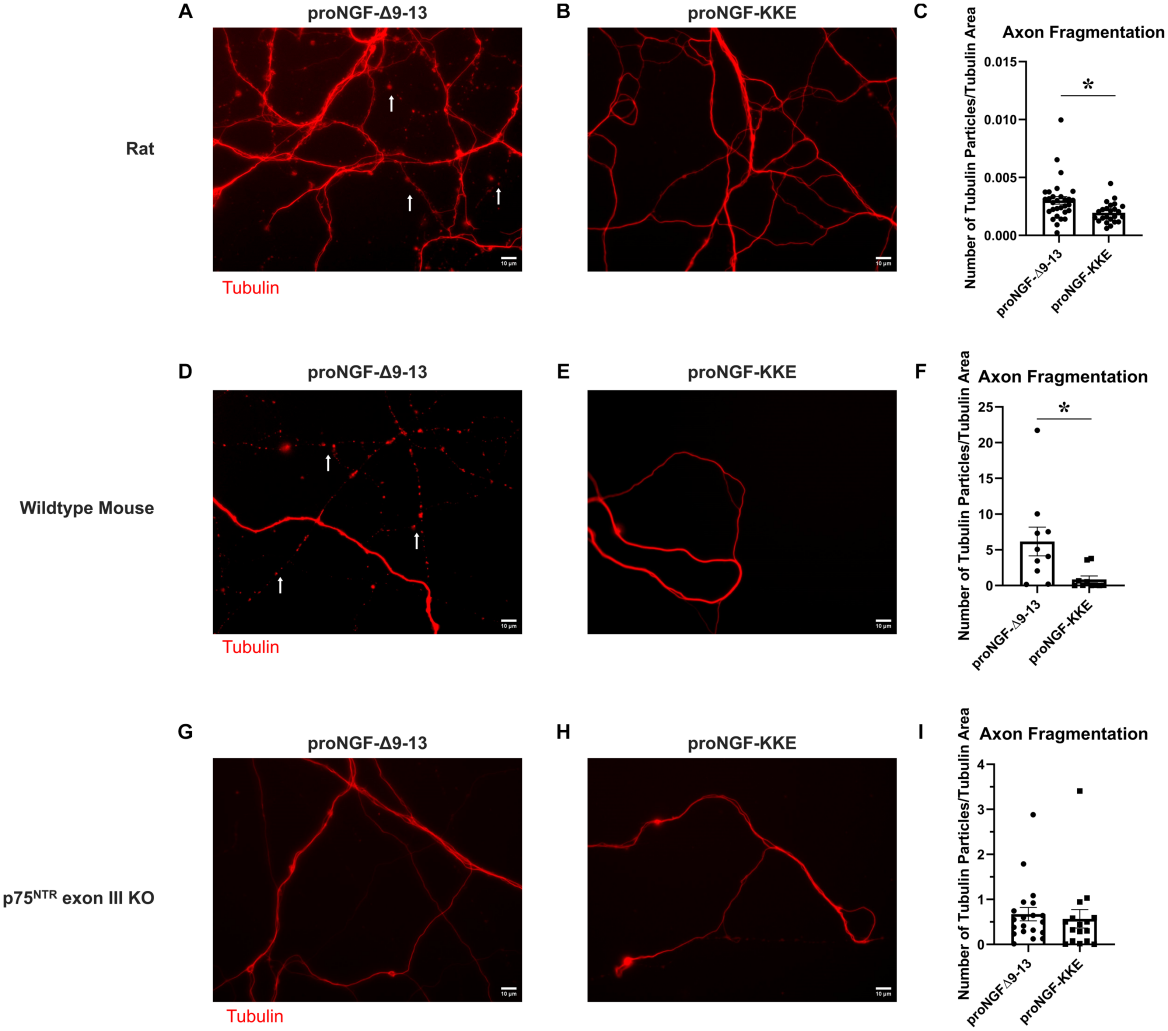
ProNGF-Δ9-13 causes greater neurodegeneration than proNGF-KKE. Aged (DIV21) rat BFCN axon terminals were incubated with either proNGF-Δ9-13 **(A)** or proNGF-KKE **(B)** for 1 h prior to imaging of tubulin (red). Tubulin images were then analyzed for fragmentation, indicative of neurodegeneration, as described in Methods. White arrows indicate areas of axon fragmentation. **(C)** Axons incubated with proNGF-Δ9-13 had a greater number of axon fragments normalized to total tubulin area compared to axons incubated with proNGF-KKE, Student’s *t*-test. For proNGF-Δ9-13, *n* = 30 images obtained from 3 chambers in 3 independent repeats. For proNGF-KKE, *n* = 24 images obtained from 3 chambers in 3 independent repeats. The highest point in the proNGF-Δ9-13 group was identified as an outlier using ROUT (Q = 1%). Removal of the outlier changed the value of p from *p* = 0.0105(*) to *p* = 0.0091(**). *p < 0.05, **p < 0.01. Error bars: SEM. DIV: days *in vitro*. **(D–I)** ProNGF-Δ9-13 or proNGF-KKE were applied to mouse p75^NTR^ exon III knockout or C57BL/6 (wildtype) BFCN axon terminals for 1 h prior to imaging of tubulin. **(D–F)** In wildtype mouse BFCNs, axons incubated with proNGF-Δ9-13 **(D)** had a greater number of axon fragments normalized to total tubulin area compared to axons incubated with proNGF-KKE **(E)**. **(F)** Quantification of axon fragmentation shown in **D** and **E.** Error bars represent SEM, *n* = 10 images per group obtained from 1 chamber each. *p < 0.05. The highest two points in the proNGF-KKE group were identified as outliers using ROUT (Q = 1%). Removal of the outliers did not affect the significance of the comparison (*p* = 0.015). **(G–I)** In p75^NTR^ exon III knockout BFCNs, no significant differences were observed in axon fragment number normalized to total tubulin area between axons incubated with proNGF-Δ9-13 **(G)** and axons incubated with proNGF-KKE **(H)**. **(I)** Quantification of axon fragmentation shown in **G** and **H.** Error bars represent SEM. For proNGF-Δ9-13, *n* = 20 images obtained from 2 chambers in 2 independent repeats. For proNGF-KKE, *n* = 16 images obtained from 2 chambers in 2 independent repeats. The highest point in each group was identified as an outlier using ROUT (Q = 1%). Removal of these outliers did not affect the significance of the comparison (*p* = 0.174). Scale bars: 10 μm.

To further assess the functional significance of the observed receptor specific proNGF transport changes in aged BFCNs, tubulin fragmentation was analyzed following incubation with either proNGF-Δ9-13 or proNGF-KKE in aged rat BFCN axon terminals. ProNGF-Δ9-13 (Figure 9A) induced greater axon fragmentation than proNGF-KKE (Figures 9B,C; *p* = 0.011). Greater axon fragmentation was also observed in wildtype (C57BL/6) mouse BFCN axons incubated with proNGF-Δ9-13 (Figure 9D) compared to those incubated with proNGF-KKE (Figures 9E,F; *p* = 0.028), confirming that these results are not specific to rat BFCNs. No differences in axon fragmentation were observed in mouse p75^NTR^ exon III knockout BFCNs incubated with either proNGF-Δ9-13 (Figure 9G) or proNGF-KKE (Figures 9H,I; *p* = 0.675), confirming that axon fragmentation occurs via p75^NTR^. These results indicate that proNGF induces neurodegeneration in aged BFCNs via p75^NTR^, suggesting that the increased proNGF-Δ9-13 transport observed in aged BFCNs causes neurodegeneration.

### 3.5. *In vitro* aging increases nitric oxide levels in rat BFCNs

**Figure 10:**
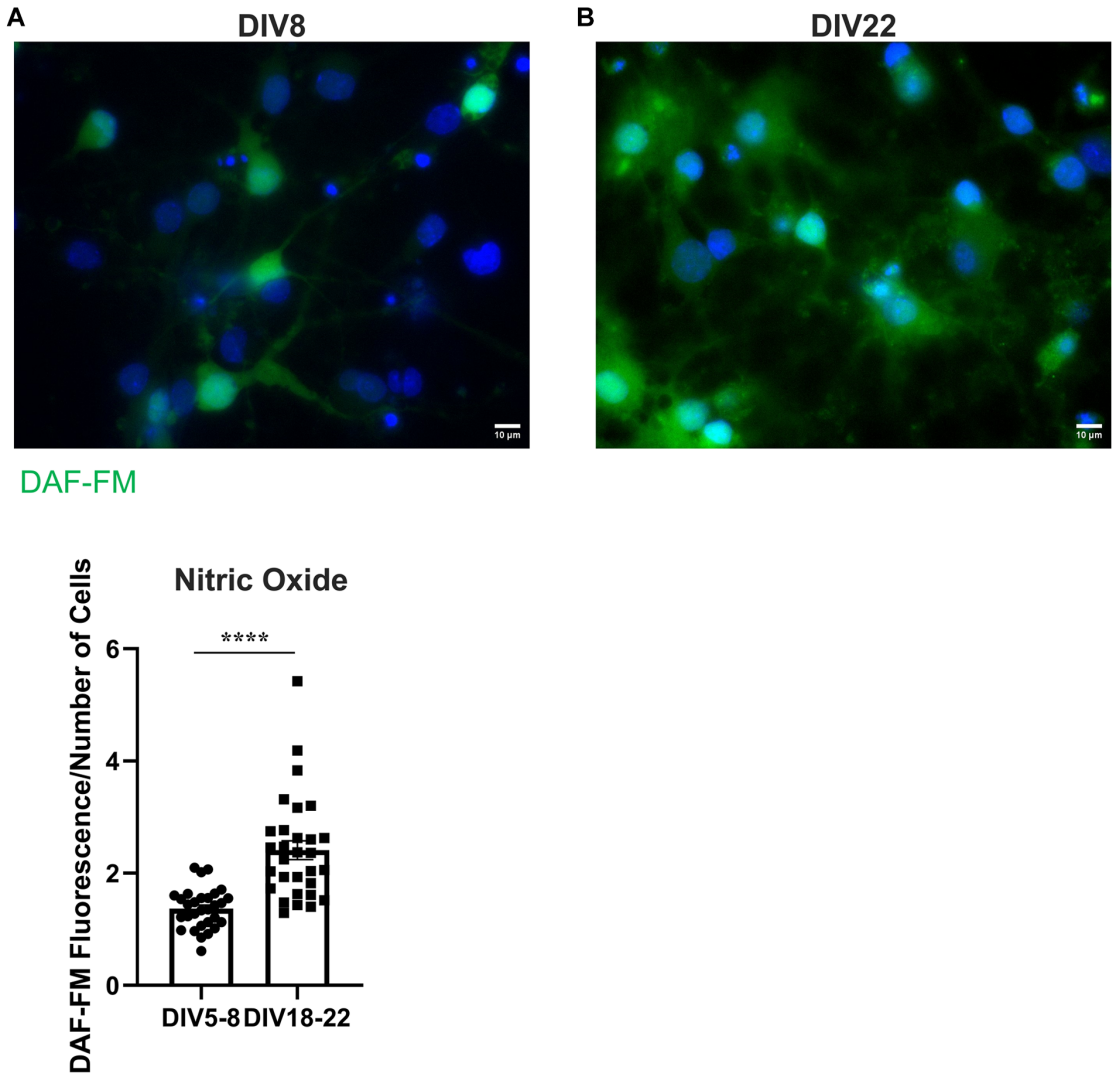
BFCNs aged *in vitro* exhibit increased nitric oxide levels compared to young BFCNs. Rat BFCNs were stained with 5 μM DAF-FM for 1 h prior to imaging the cell bodies. Aged BFCNS [**(B)**, DIV18-22] exhibit increased DAF-FM fluorescence (green) relative to young BFCNs [**(A)**, DIV5-8], indicating that nitric oxide levels increase with *in vitro* age. Nuclei are labeled with DAPI (blue). **(C)** Quantification of the images shown in **A,B**, Student’s *t*-test. *n* = 30 images/group from three chambers in three independent experiments. The highest point in the DIV18-22 group was identified as an outlier using ROUT (Q = 1%). Removal of the outlier did not change the significance of the comparison. Error bars represent SEM. ****p < 0.0001. DIV, days *in vitro*. Scale bars: 10 μm.

To determine whether nitrative stress may contribute to the observed receptor specific proNGF transport deficits, we investigated whether reactive nitrogen species levels increase with age *in vitro* as they do *in vivo*. NO levels were assessed in aged (DIV18-22) and young (DIV5-8) rat BFCNs by staining with 5 μM DAF-FM, a fluorescent stain that detects NO. DAF-FM fluorescence was increased in aged BFCN soma (Figure 10B) compared to young BFCN soma (Figures 10A,C; *p* < 0.0001), indicating that NO levels increase with *in vitro* age.

### 3.6. Nitrative stress increases retrograde transport of proNGF via p75^NTR^ but decreases retrograde transport of proNGF via TrkA

**Figure 11:**
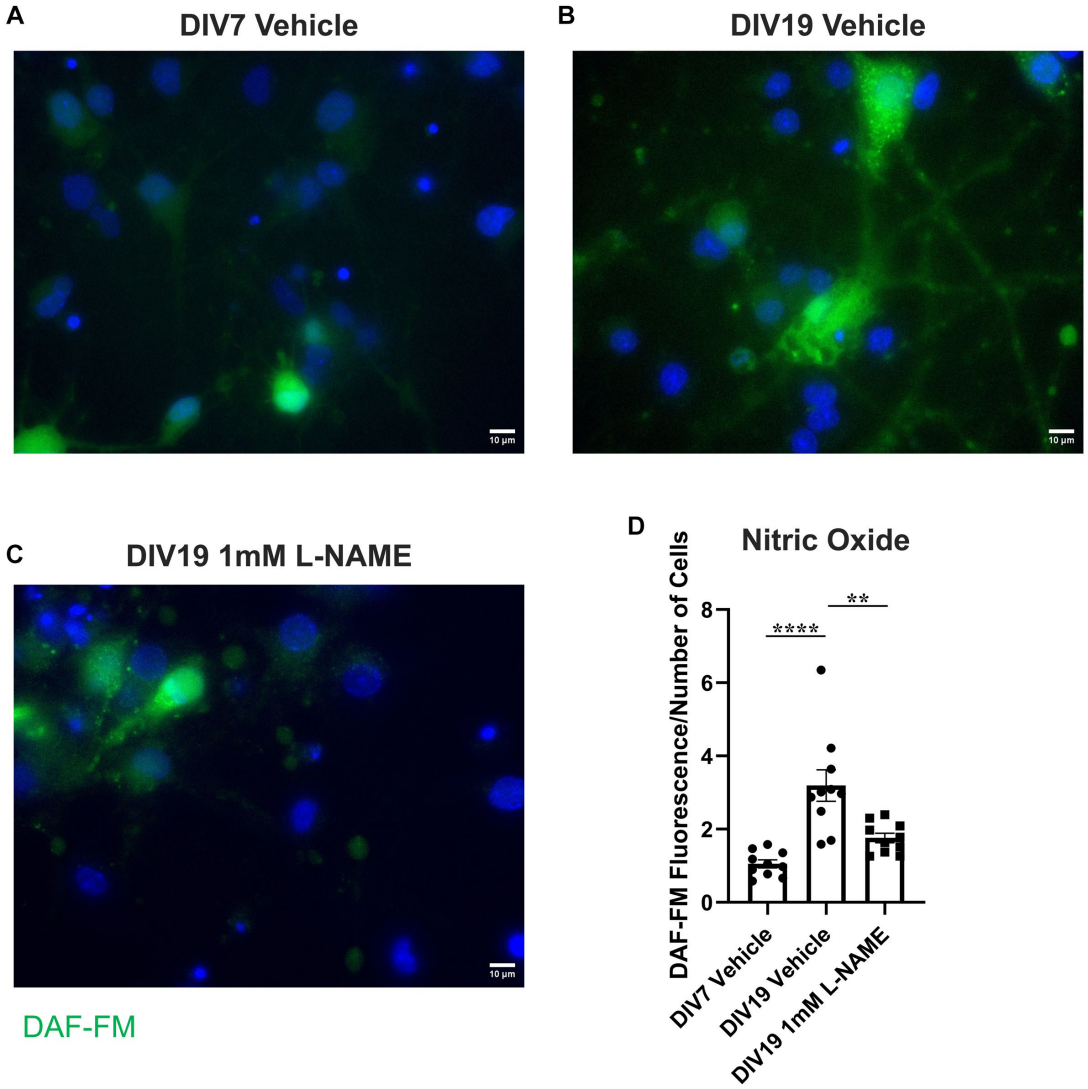
L-NAME reduces nitric oxide levels in aged BFCNs. Rat BFCNs were treated with either 1 mM L-NAME or vehicle for 24 h prior to staining with 5 μM DAF-FM to detect nitric oxide levels in the cell bodies. **(A–C)** Aged (DIV19) BFCNs treated with 1 mM L-NAME for 24 h **(C)** exhibited reduced DAF-FM fluorescence (green) compared to aged BFCNs treated with vehicle **(B)**. L-NAME treatment restored DAF-FM fluorescence to the levels seen in young (DIV7) vehicle-treated BFCNs **(A)**. DAPI is shown in blue. **(D)** Quantification of the images shown in **A–C**, one-way ANOVA and *post hoc* Tukey test. *N* = 10 images/group. For each group, images were obtained from one chamber. No outliers were identified using ROUT (Q = 1%). Error bars represent SEM. **p < 0.01, ****p < 0.0001. DIV, days *in vitro*. Scale bars: 10 μm.

To determine whether the observed increases in nitric oxide contribute to age-induced receptor specific proNGF transport changes, aged rat BFCNs were treated with 1 mM L-NAME, an inhibitor of nitric oxide synthase, for 24 h prior to analysis of proNGF-Δ9-13 and proNGF-KKE retrograde transport. L-NAME treatment of aged rat BFCNs (Figure 11C) reduced DAF-FM fluorescence compared to aged vehicle-treated BFCNs (Figures 11B,D; *p* = 0.002), validating that NO levels were reduced following L-NAME treatment. DAF-FM fluorescence was not significantly different in aged BFCNs treated with L-NAME (Figure 11C) compared to young vehicle-treated BFCNs (Figures 11A,D; *p* = 0.168), indicating that L-NAME normalized the elevated NO levels in aged BFCNs.

**Figure 12:**
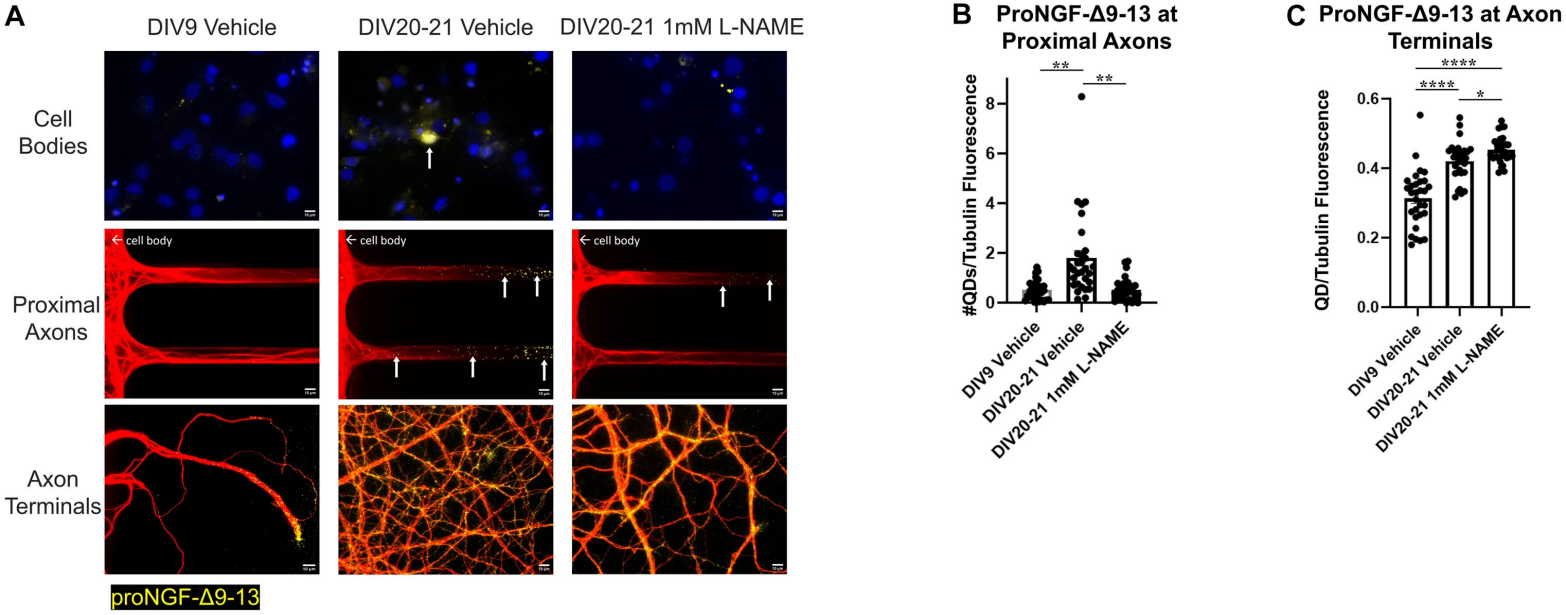
Nitrative stress increases retrograde transport of proNGF-Δ9-13 in aged BFCNs. Rat BFCNs were treated with either 1 mM  L-NAME or vehicle for 24 h prior to analysis of proNGF-Δ9-13 retrograde transport. ProNGF-Δ9-13 is shown in yellow, tubulin is shown in red, and DAPI is shown in blue. Yellow arrows indicate quantum dots that have accumulated in cell bodies or proximal axons. **(A)** ProNGF-Δ9-13 accumulated in aged BFCNs treated with vehicle but not in young BFCNs treated with vehicle or aged BFCNs treated with L-NAME. Aged BFCNs (DIV20-21) treated with 1 mM  L-NAME for 24 h exhibited decreased accumulation of proNGF-Δ9-13 at the cell bodies and the proximal axons [top and middle rows, quantification in **(B)**] and increased accumulation of proNGF-Δ9-13 at the axon terminals [bottom row, quantification in **(C)**] relative to aged-vehicle treated controls. Aged BFCNs treated with L-NAME showed no difference in accumulation of proNGF-Δ9-13 at cell bodies and he proximal axons [top and middle rows, quantification in **(B)**] compared to young vehicle-treated controls. Aged BFCNs exhibited greater accumulation of proNGF-Δ9-13 at the axon terminals relative to young (DIV9) vehicle-treated controls regardless of treatment condition [bottom row, quantification in **(C)**]. Tubulin is shown in red, and proNGF-Δ9-13 is shown in yellow. **(B,C)** Quantification of the images shown in **A**. *N* = 30 images/group at both the proximal axons **(B)** and axon terminals **(C)**. All sample sizes were generated from three chambers in three independent experiments. At the proximal axons, the highest point in the DIV20-21 vehicle group was detected as an outlier using ROUT (Q = 1%). Removal of this outlier changed the comparisons between this group and both the DIV7-9 vehicle and the DIV20-21  L-NAME group from *p* < 0.01 (**) to *p* < 0.001 (***). One-way ANOVA and *post hoc* Tukey test, *p < 0.05, **p < 0.01, ****p < 0.0001. DIV, days *in vitro*; QD, quantum dot. Scale bars: 10 μm.

**Figure 13:**
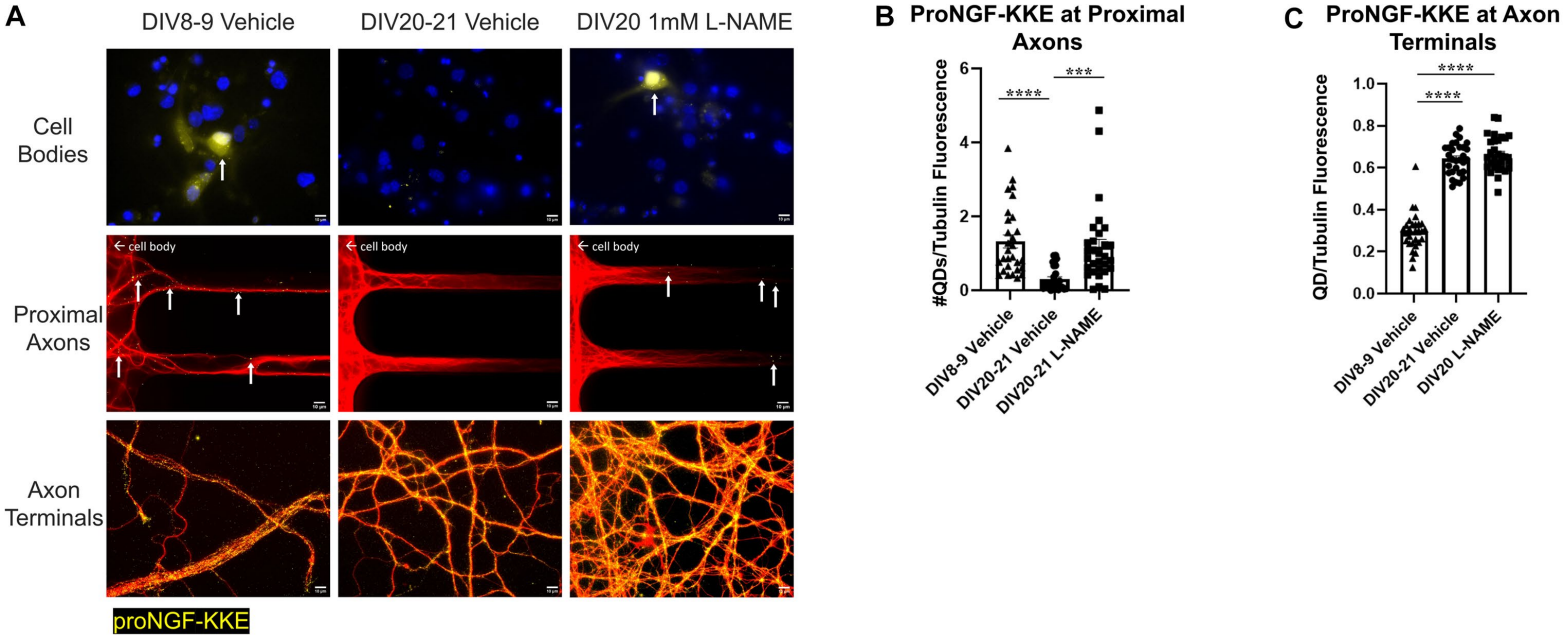
Nitrative stress decreases retrograde transport of proNGF-KKE in aged BFCNs. Rat BFCNs were treated with either 1 mM  L-NAME or vehicle for 24 h prior to analysis of proNGF-KKE retrograde transport. ProNGF-KKE is shown in yellow, tubulin is shown in red, and DAPI is shown in blue. Yellow arrows indicate quantum dots that have accumulated in cell bodies or proximal axons. **(A)** ProNGF-KKE accumulated in young BFCNs treated with vehicle and aged BFCNs treated with L-NAME but not in aged BFCNs treated with vehicle. Aged BFCNs (DIV20-21) treated with 1 mM  L-NAME for 24 h exhibited increased accumulation of proNGF-KKE at the cell bodies and proximal axons [top and middle rows, quantification in **(B)**] relative to aged vehicle-treated controls. Aged BFCNs had greater proNGF-KKE accumulation at the axon terminals greater proNGF-KKE accumulation at the axon terminals [bottom row, quantification in **(C)**] relative to young (DIV8-9) vehicle-treated controls, regardless of treatment condition. Tubulin is shown in red, and proNGF-KKE is shown in yellow. **(B,C)** Quantification of the images shown in **A**. **(B)** Proximal axons: DIV8-9 vehicle *n* = 29 images, DIV20-21 vehicle *n* = 28 images, DIV20 L-NAME *n* = 30 images. The highest three points of the DIV20-21 vehicle, the highest two points of the DIV20 L-NAME, and the highest point of the DIV8-9 vehicle were detected as outliers using ROUT (Q = 1%). Removal of these outliers did not affect the significance of the comparison between DIV20 L-NAME and DIV8-9 vehicle (*p* = 0.2618) or between DIV20-21 vehicle and DIV8-9 vehicle (*p* < 0.0001). Removal of the outliers changed the comparison between DIV20-21 vehicle and DIV20 L-NAME groups from *p* = 0.0004 (***) to *p* < 0.0001 (****). **(C)** Axon terminals: DIV8-9 vehicle *n* = 29 images, DIV20-21 vehicle *n* = 30 images, DIV20 L-NAME *n* = 30 images. The highest point in the DIV8-9 group was detected as an outlier using ROUT (Q = 1%). Removal of this outlier did not affect the observed value of *p*. All sample sizes were generated from three chambers in three independent experiments. One-way ANOVA and *post hoc* Tukey test, ***p < 0.001, ****p < 0.0001. DIV, days *in vitro*; QD, quantum dot. Scale bars: 10 μm.

L-NAME treatment decreased retrograde transport of proNGF-Δ9-13 in aged rat BFCNs, indicated by decreased proNGF-Δ9-13 accumulation in the cell bodies and at the proximal axons (Figure 12A top and middle rows, Figure 12B; *p* = 0.001) compared to aged vehicle-treated controls. Aged BFCNs treated with L-NAME exhibited no difference in accumulation of proNGF-Δ9-13 at the proximal axons compared to young vehicle-treated controls (Figure 12A top and middle rows, Figure 13B; *p* > 0.999), indicating that inhibition of nitric oxide production normalized the age-induced increase in transport of proNGF bound to p75^NTR^. Interestingly, L-NAME increased the level of proNGF-Δ9-13 at the axon terminals compared to both aged vehicle-treated BFCNs (Figure 12A bottom row, Figure 12C; *p* = 0.026) and young vehicle-treated BFCNs (Figure 12A bottom row, Figure 12C; *p* < 0.0001).

Conversely, rat BFCNs treated with L-NAME exhibited increased accumulation of proNGF-KKE at the cell bodies and proximal axons compared to aged vehicle-treated BFCNs (Figure 13A top and middle rows, Figure 13B; *p* = 0.0004), indicating that L-NAME increased retrograde transport of proNGF-KKE. No differences in the accumulation of proNGF-KKE at the cell bodies or proximal axons were observed between aged BFCNs treated with L-NAME and young BFCNs treated with vehicle (Figure 13A top and middle rows, Figure 13B; *p* = 0.582), indicating that reducing nitric oxide fully rescued age-induced deficits in retrograde transport of proNGF-KKE. Aged BFCNs had greater levels of proNGF-KKE at axon terminals relative to young BFCNs regardless of treatment condition (Figure 13A bottom row, Figure 13C; DIV20-21 vehicle vs. DIV8-9 vehicle *p* < 0.0001, DIV20 L-NAME vs. DIV8-9 vehicle *p* < 0.0001). No differences were observed in levels of proNGF-KKE at the axon terminals in aged BFCNs treated with L-NAME or vehicle (Figure 13A bottom row, Figure 13C; *p* = 0.724). These results suggest that the increased proNGF-KKE transport observed with L-NAME treatment is not due to changes in binding at the axon terminals.

### 3.7. Nitrative stress does not alter TrkA or p75^NTR^ levels

**Figure 14:**
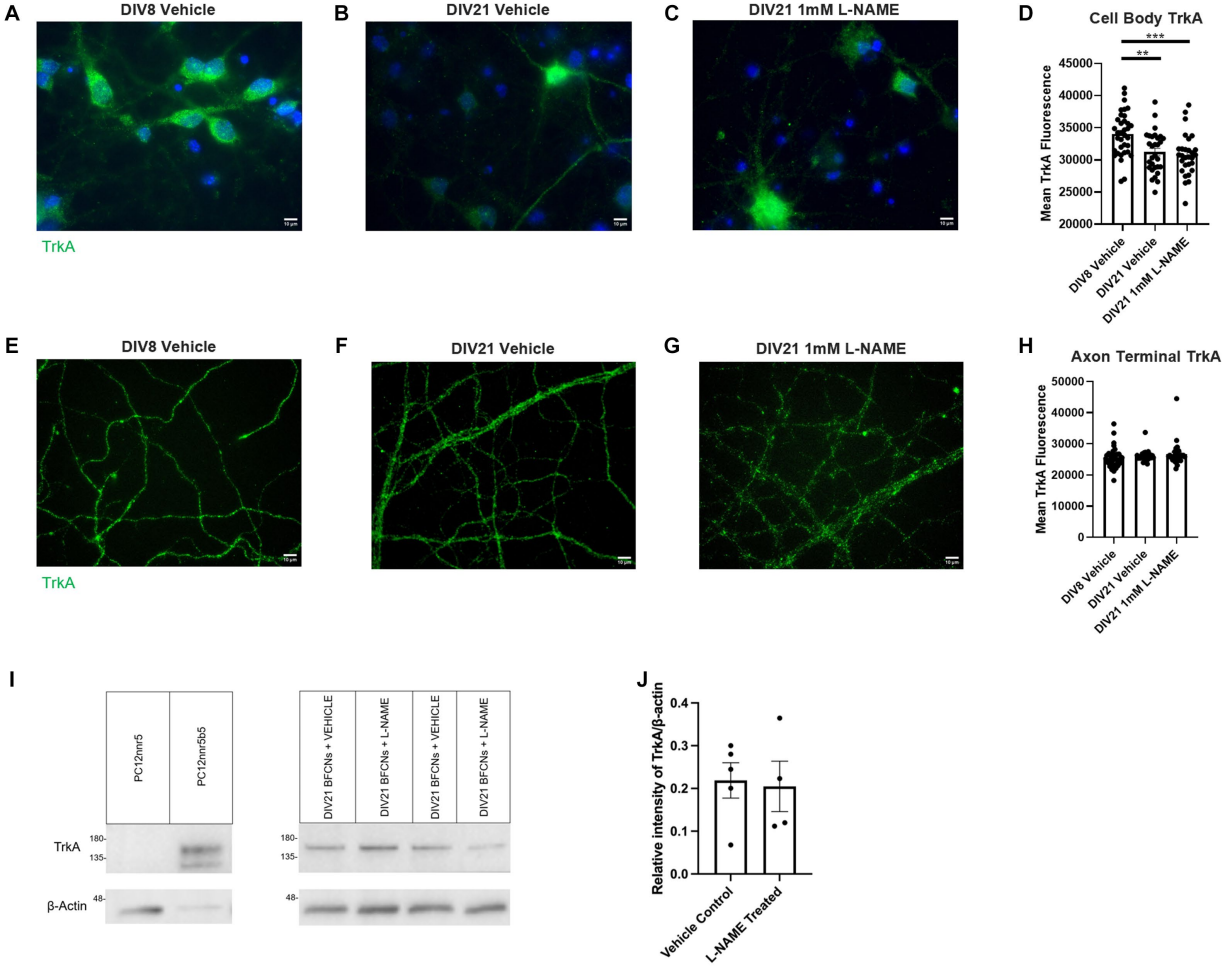
L-NAME treatment does not affect TrkA levels in aged BFCNs. Aged rat BFCNs (DIV21) were treated with 1 mM L-NAME for 24 h prior to analysis of TrkA levels using immunocytochemistry. TrkA is shown in green and DAPI is shown in blue. **(A,E)** DIV8 BFCNs treated with vehicle; **(B,F)** DIV21 BFCNs treated with vehicle; **(C,G)** DIV21 BFCNs treated with L-NAME; **(D)** Quantification of the images shown in **A–C**. TrkA levels at the cell bodies were decreased in aged BFCNs relative to young BFCNs regardless of treatment condition. No differences in TrkA levels were observed between aged BFCNs treated with L-NAME and aged vehicle-treated control BFCNs. For the DIV8 vehicle, *n* = 35 images, DIV21 vehicle *n* = 30 images, DIV21 L-NAME *n* = 29 images. One-way ANOVA and *post hoc* Tukey test, **p < 0.01, ***p < 0.001, **(H)** Quantification of the images shown in **E–G**. No differences in TrkA levels were observed between any of the groups at the axon terminals. For the DIV8 vehicle-treated group, *n* = 35 images, for both aged groups, *n* = 30 images. All images were obtained from three chambers in three independent experiments. One-way ANOVA, *p* > 0.05. The highest point in each group was detected as an outlier using ROUT (Q = 1%). Removal of these outliers did not affect the significance of the comparison (*p* = 0.182). Error bars: SEM. DIV, days *in vitro*. **(I,J)** Immunocytochemistry results were confirmed using western blotting for TrkA in DIV21 BFCNs treated with either vehicle or 1 mM L-NAME. **(I)** Representative western blot comparing relative intensity of TrkA expression normalized to β-actin expression. 16.8 μg of total protein was loaded in each BFCN sample well; 5.88 μg protein for PC12nnr5 and 1.68 μg protein for PC12nnr5B5 were loaded as negative and positive controls, respectively. **(J)** There were no differences in TrkA expression between BFCNs treated with L-NAME (*n* = 4) and vehicle treatment (*n* = 5; *p* = 0.85). Error bars: SEM. DIV, days *in vitro*. Scale bars: 10 μm.

To further investigate the mechanisms by which L-NAME increases retrograde transport of proNGF-KKE and decreases retrograde transport of proNGF-Δ9-13, TrkA and p75^NTR^ levels were analyzed using immunocytochemistry in aged rat BFCNs following L-NAME treatment. TrkA levels were decreased in aged BFCN cell bodies relative to young BFCNs regardless of treatment condition (Figures 14A–D, aged vehicle vs. young vehicle *p* = 0.003, aged L-NAME vs. young vehicle *p* = 0.0004). L-NAME did not affect TrkA levels at the cell bodies compared to aged vehicle-treated BFCNs (Figures 14B–D; *p* = 0.85). No differences were observed between any of the groups at the axon terminals (Figures 14E–H; *p* = 0.301). These results were confirmed by western blotting (Figure 14I). No differences in TrkA levels were observed between DIV21 BFCNs treated with vehicle or L-NAME (Figure 14J; *p* = 0.85).

**Figure 15:**
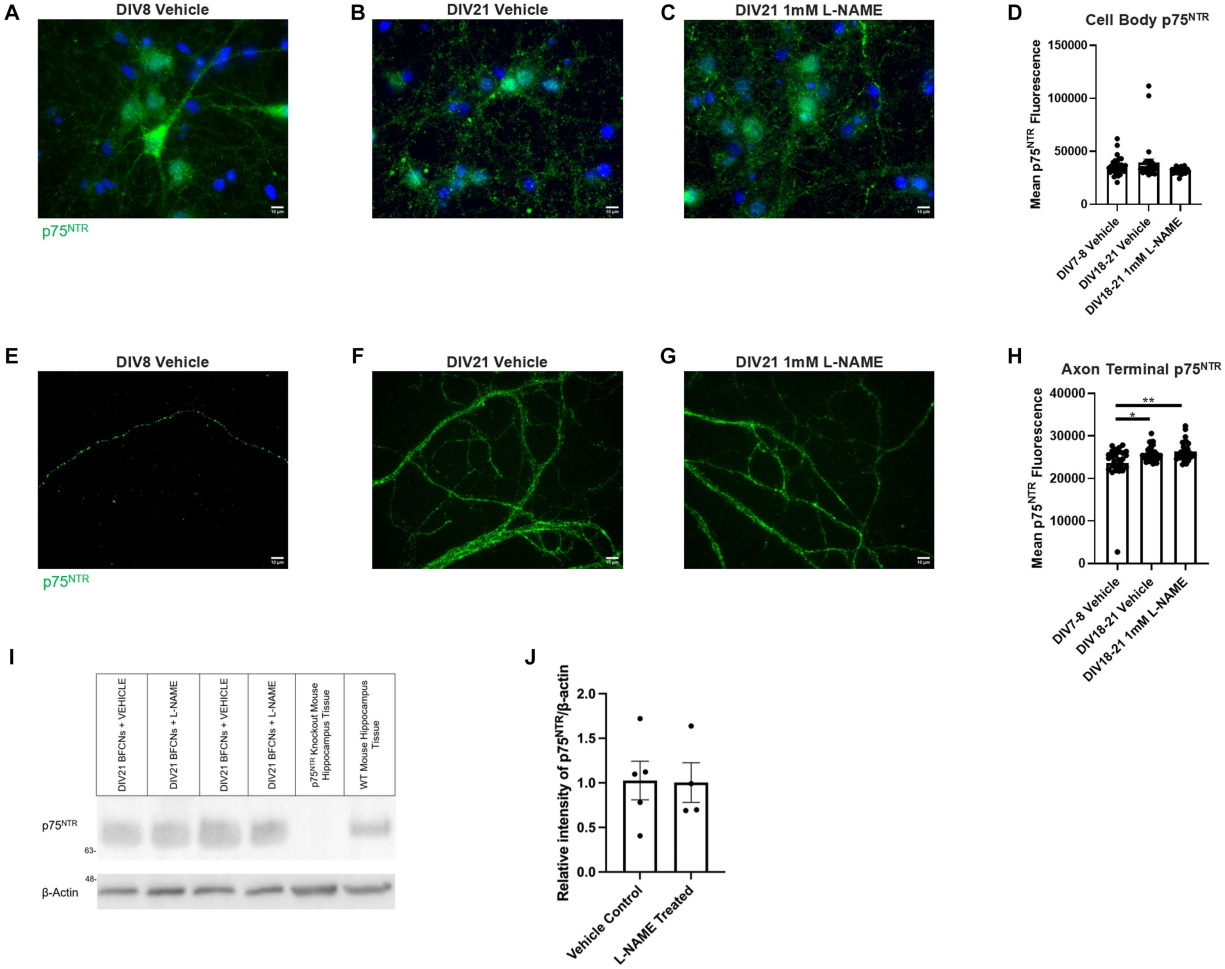
L-NAME treatment does not affect p75^NTR^ levels in aged BFCNs. Aged rat BFCNs (DIV18-21) were treated with 1 mM  L-NAME for 24 h prior to analysis of p75^NTR^ levels using immunocytochemistry. p75^NTR^ is shown in green and DAPI is shown in blue. **(A,E)** DIV8 BFCNs treated with vehicle; **(B,F)** DIV21 BFCNs treated with vehicle; **(C,G)** DIV21 BFCNs treated with L-NAME; **(D)** Quantification of the images shown in **A–C**. No differences in p75^NTR^ levels were observed between any of the groups at the cell bodies. For both vehicle-treated groups, *n* = 30 images. For L-NAME, *n* = 29 images. For each group, images were obtained from three chambers in three independent experiments. One-way ANOVA, *p* > 0.05. The highest two points of the DIV18-21 vehicle and the highest point of the DIV7-9 vehicle were detected as outliers using ROUT (Q = 1%). Removal of these outliers did not change the significance of the comparison (*p* = 0.061). **(H)** Quantification of the images shown in **E–G**. Aged BFCNs had greater p75^NTR^ levels at the axon terminals compared to young BFCNs regardless of treatment condition. For all groups, *n* = 30 images obtained from three chambers in three independent experiments. The lowest point of the DIV7-9 vehicle was detected as an outlier using ROUT (Q = 1%). Removal of this outlier did not affect the significance of the comparison between the vehicle-treated groups (*p* = 0.01) but changed the comparison between DIV7-8 vehicle and DIV18-21  L-NAME from *p* = 0.002 to *p* = 0.0006. One-way ANOVA and *post hoc* Tukey test, *p < 0.05, **p < 0.01. Error bars: SEM. DIV, days *in vitro*. **(I,J)** Immunocytochemistry results were confirmed using western blotting for p75^NTR^ in DIV21 BFCNs treated with either vehicle or 1 mM  L-NAME. **(I)** Representative western blot comparing relative intensity of p75^NTR^ expression normalized to β-actin expression. 16.8  μg of total protein was loaded in each BFCN sample well; 33.6  μg total protein from p75^NTR^ exon III knockout and wildtype mouse hippocampal tissues were loaded as negative and positive controls, respectively. **(J)** There were no differences in p75^NTR^ expression between BFCNs treated with L-NAME (*n* = 4) and vehicle treatment (*n* = 5; *p* = 0.95). Error bars: SEM. DIV, days *in vitro*. Scale bars: 10 μm.

No differences in p75^NTR^ levels were observed between groups at the cell bodies (Figures 15A–D, *p* = 0.072). At the axon terminals, p75^NTR^ levels were increased in aged BFCNs relative to young BFCNs regardless of treatment condition (Figures 15E,F,H, DIV7-8 vehicle vs. DIV18-21 vehicle *p* = 0.013; Figures 15E,G,H, DIV7-8 vehicle vs. DIV18-21 L-NAME *p* = 0.002,). No differences in p75^NTR^ levels at the axon terminals were observed between DIV18-21 BFCNs treated with vehicle or L-NAME (Figures 15F–H; *p* = 0.819). These results were confirmed by western blotting (Figure 15I). No differences in p75^NTR^ levels were observed between DIV21 BFCNs treated with vehicle or L-NAME (Figure 15J; *p* = 0.95).

## 4. Discussion

These findings indicate that *in vitro* aging and nitrative stress decrease retrograde transport of proNGF bound to TrkA while increasing retrograde transport of proNGF bound to p75^NTR^. The increased retrograde transport of proNGF-Δ9-13 and the concurrent decrease in retrograde transport of proNGF-KKE in aged BFCNs are associated with increased apoptotic signaling, decreased neurotrophic signaling, and increased neurodegeneration. These results are supported by previous studies showing that proNGF induces apoptotic signaling when TrkA is reduced (Lee, 2001; Nykjaer et al., 2004; Masoudi et al., 2009; Ioannou and Fahnestock, 2017). Although proNGF transport was analyzed following a 1 h axon terminal incubation with proNGF-Δ9-13 or proNGF-KKE, signaling effects were observed after axon terminal proNGF incubation for only 15 min. These findings are supported by literature indicating that signaling factors are rapidly activated in cell bodies after stimulation of fluidically isolated axon terminals with neurotrophic factors. For example, activation of CREB, a transcription factor activated downstream of the ERK signaling pathway, occurs in cell bodies 10 min after axonal incubation with NGF (Riccio et al., 1997). Similarly, activated Trk was observed in neuronal cell bodies 1 min after NGF was applied to axon terminals (Senger and Campenot, 1997). In the latter study, activated Trk and other active signaling factors were observed in cell bodies prior to accumulation of NGF, suggesting that propagation of signaling cascades occurs faster than retrograde transport of the neurotrophin-receptor complex (Senger and Campenot, 1997). Together, these studies suggest that neurotrophin signaling promotes survival of cell bodies while axonal transport promotes maintenance of axons and synapses. The observed receptor-specific transport deficits and the resulting signaling alterations may contribute to loss of BFCN axons and synapses in aging, as ERK and Akt signaling downstream of TrkA are essential for neurite outgrowth and cell survival and target-derived neurotrophins are critical for axon maintenance (Klesse et al., 1999; Kaplan and Miller, 2000; Cosker et al., 2013).

L-NAME, a nitric oxide synthase inhibitor, rescued deficits in TrkA-dependent proNGF transport and normalized excessive proNGF transport via p75^NTR^, suggesting that nitrative stress downstream of NO contributes to receptor-specific retrograde transport deficits. Under healthy conditions, NO is non-toxic and is an essential signaling factor involved in important physiological processes such as blood flow regulation, immune system function, and release of acetylcholine from cholinergic neurons (Prast and Philippu, 2001; Pacher et al., 2007). However, when NO is present in high levels, it may produce toxic species. For example, NO can react with superoxide to form the toxic reactive nitrogen species, ONOO^−^ (Pacher et al., 2007). SIN-1, a ONOO^−^ generator, has previously been shown to reduce TrkA activation and downstream signaling in PC12 cells (Jonnala and Buccafusco, 2001). ONOO^−^ also has been shown to cause nitrative modification of NGF, leading to increased apoptotic activity via p75^NTR^ (Pehar et al., 2006). Although the toxic species in our study cannot be identified with certainty, our results support the observation that the effects of nitrative stress on proNGF transport are different for TrkA vs. p75^NTR^.

L-NAME did not affect levels of TrkA or p75^NTR^ in aged BFCNs, suggesting that the mechanism by which nitrative stress decreases retrograde transport of proNGF via TrkA and increases retrograde transport of proNGF via p75^NTR^ is not by changing receptor levels. Our results are supported by literature indicating that TrkA levels in the diabetic retina, where nitrative stress is increased, do not differ from healthy controls (Ali et al., 2008). These findings are consistent with previous literature indicating that reactive nitrogen species and nitration of TrkA inhibit TrkA activation and signaling (Jonnala and Buccafusco, 2001; Ali et al., 2008; Kropf and Fahnestock, 2021). Nitrative stress may also differentially affect retrograde transport of proNGF via TrkA and p75^NTR^ by proNGF nitration. Nitration of NGF by peroxynitrite has been shown to induce neuronal death via p75^NTR^ and reduce its ability to activate TrkA (Jonnala and Buccafusco, 2001; Pehar et al., 2006; Bruno et al., 2009). Nitrated proNGF is observed in the Alzheimer’s disease (AD) brain, indicating that studies on NGF nitration are also relevant to the precursor form (Bruno et al., 2009). Together, these results suggest that nitrative stress may alter the affinity of proNGF for its receptors, increasing proNGF binding to p75^NTR^ while decreasing its binding to and activation of TrkA. Future studies that investigate how nitrative stress affects the binding of proNGF to each of its receptors will be important for elucidating this mechanism.

Our observation that proNGF transport via p75^NTR^ is increased in aged BFCNs is supported by previous work indicating that accumulation of proNGF in the cortex results in internalization of p75^NTR^ in vesicles (Allard et al., 2018). Because NGF-p75^NTR^ is internalized in signaling endosomes for retrograde transport, it is possible that the vesicular p75^NTR^ observed in the previous study was subsequently transported (Bronfman et al., 2003). Interestingly, although our results indicate that retrograde transport of proNGF bound to p75^NTR^ is increased in aged BFCNs, *in vivo* studies indicate that proNGF transport is reduced in the aged brain (Cooper et al., 1994). This discrepancy may be due to the amount of time that our BFCNs aged *in vitro* were exposed to proNGF-Δ9-13. In the present study, aged BFCN axons were exposed to proNGF-Δ9-13 for 1 h, which produced axon fragmentation indicative of neurodegeneration but was not sufficient to prevent retrograde transport. Longer incubations with proNGF-Δ9-13 may cause greater axon degeneration, preventing retrograde transport. Our finding in aged neurons that proNGF-Δ9-13 induces greater axon fragmentation than proNGF-KKE is supported by previous literature indicating that activation of p75^NTR^ in the absence of TrkA may cause neurodegeneration, as it inhibits neurite outgrowth and induces growth cone collapse, while activation of TrkA induces opposing effects (Gallo and Letourneau, 2004; Yamashita et al., 2005; Deinhardt et al., 2011). Such axonal degeneration may prevent proNGF from being transported via p75^NTR^ in the aged brain. Short-term exposure to proNGF may increase proNGF transport via p75^NTR^ and activation of its JNK signaling cascade, whereas prolonged exposure to proNGF and p75^NTR^-induced activation of JNK signaling in the aged brain, where TrkA is reduced (Cooper et al., 1994; Terry et al., 2011), may contribute to BFCN axonal degeneration, preventing further transport.

A limitation of this study is that the inability of proNGF-Δ9-13 to bind to TrkA is not a completely accurate representation of the conditions in the aged brain. While TrkA levels are reduced in BFCNs aged *in vitro* and *in vivo*, TrkA is not completely eliminated (Cooper et al., 1994; Terry et al., 2011; Shekari and Fahnestock, 2019). The low levels of remaining TrkA may reduce proNGF binding to p75^NTR^ and subsequent retrograde transport in aged BFCNs *in vivo* and *in vitro*.

Prior to retrograde transport, the NGF-TrkA complex is internalized at axon terminals via both clathrin-dependent and pincher-dependent mechanisms (Howe et al., 2001; Shao et al., 2002). In this study, the increased levels of proNGF-KKE and proNGF-Δ9-13 in aged BFCN axon terminals relative to those seen in young BFCNs indicates that this internalization process is conserved with *in vitro* age. These results, along with decreased proNGF accumulation at proximal axons, suggest that proNGF transport deficits are related to axonal transport machinery rather than internalization machinery. Indeed, clathrin-mediated internalization machinery increases in both aged human brain and rodent neurons aged *in vitro* (Alsaqati et al., 2018; Burrinha et al., 2021), indicating that aging enhances endocytosis (Alsaqati et al., 2018; Burrinha et al., 2021). In contrast, the function of the retrograde transport motor, dynein, is decreased with age, as it accumulates in nerve endings and exhibits decreased interaction with dynactin, a co-factor necessary for retrograde transport (Waterman-Storer et al., 1997; Kimura et al., 2007). Together, these studies suggest that the observed accumulation of proNGF in aged BFCN axon terminals may occur due to an increased rate of endocytosis along with decreased retrograde transport. The literature reports conflicting effects of nitrative stress on endocytosis. NO has been shown to increase receptor-mediated endocytosis via s-nitrosylation of dynamin, enhancing its GTPase activity (Lowenstein, 2007). NO also enhances endocytosis in hippocampal neurons by a cGMP-dependent mechanism (Micheva et al., 2003). In other studies, however, NO had an inhibitory effect on endocytosis of synaptic vesicles (Yakovleva et al., 2013) and on mannan by reducing binding to its receptor (Martinez-Zubiaurre et al., 1996). In the present study, however, it is unlikely that nitrative stress significantly altered endocytosis of proNGF in aged BFCNs, as L-NAME had very little effect on the amount of proNGF-Δ9-13 or proNGF-KKE at aged BFCN axon terminals. The increased levels of proNGF-KKE and proNGF-Δ9-13 at axon terminals in aged BFCNs may therefore be due to increased levels of clathrin-mediated internalization machinery, as previously reported in the aged brain (Alsaqati et al., 2018).

Our observation that proNGF-KKE levels are higher in aged BFCN axon terminals relative to young BFCN axon terminals indicates that the observed decrease in proNGF-KKE accumulation at the proximal axons in aged neurons is not caused by neuron loss. If the aged chambers contained fewer neurons, one would also expect to see decreased accumulation at the axon terminals relative to young BFCNs. Our findings that p75^NTR^ levels are not different between young and aged BFCNs are further evidence for consistent BFCN numbers across *in vitro* ages. These findings are consistent with previous literature showing that p75^NTR^ levels remain unchanged in BFCNs aged *in vitro* and *in vivo* (Cooper et al., 1994; Shekari and Fahnestock, 2019).

Aging is the greatest risk factor for developing AD and other neurodegenerative disorders (Yankner et al., 2008; Tönnies and Trushina, 2017). ProNGF transport deficits and receptor imbalances are also observed in AD, which likely contribute to the BFCN degeneration that occurs in early stages of the disease (Ernfors et al., 1990; Cooper et al., 1994; Mufson et al., 1996, 1997; Hock et al., 1998; Peng et al., 2004; Podlesniy et al., 2006; Lee et al., 2009; Salehi et al. 2006; Perez et al., 2011; Fahnestock and Shekari, 2019). The increased nitrative stress levels that occur in aging are further exacerbated in mild cognitive impairment and AD (Tohgi et al., 1999; Zhang et al., 2005; Butterfield et al., 2011). The results of this study may therefore also be relevant to AD and other age-related neurodegenerative diseases.

In conclusion, our findings indicate that retrograde transport of proNGF via TrkA is impaired while transport of proNGF via p75^NTR^ is enhanced in aged BFCNs. Nitrative stress contributes to these receptor-specific transport alterations, which are associated with increased apoptotic signaling, decreased neurotrophic signaling, and neurodegeneration. This study improves understanding of the receptor specificity of proNGF transport deficits that occur in aged BFCNs and identifies a contributing mechanism. These findings are critical for understanding why BFCNs lose function with age, which is associated with neurodegeneration and cognitive decline.

## Data availability statement

The datasets presented in this study can be found in online repositories. The names of the repository/repositories and accession number(s) can be found at: Name of repository: McMaster Dataverse. Accession number: https://borealisdata.ca/dataset.xhtml?persistentId=doi:10.5683/SP3/SQIEFO.

## Ethics statement

The animal study was approved by McMaster University Animal Research Ethics Board. The study was conducted in accordance with the local legislation and institutional requirements.

## Author contributions

EK, AS, and MF contributed to the conception and design of the study. EK, AS, AP, and SJ performed experiments and analyzed the data. CW contributed essential methods and resources. EK wrote the manuscript. AS, AP, and SJ wrote sections of the manuscript. All authors contributed to manuscript revision, read, and approved the submitted version.

## Supplementary material

SUPPLEMENTARY FIGURE 1 Boiled proNGF is inactive. Heat inactivated proNGF or vehicle were applied to rat BFCN axon terminals for 15 minutes, followed by quantification of signaling factor activation at BFCN cell bodies. **A)** Inactivated proNGF and vehicle induced the same level of JNK activation, both of which induced lower JNK activation than proNGF-Δ9-13. **B)** Inactivated proNGF and vehicle induced the same level of ERK activation, both of which induced lower ERK activation than proNGF-KKE. Error bars represent SEM. For inactivated proNGF and vehicle, n=10 images/group taken from 1 chamber. For proNGF-Δ9-13 and proNGF-KKE, n=30 images/group taken from 3 chambers.
